# Genome-Wide Characterization and Expression Analysis of the HD-ZIP Gene Family in Response to Salt Stress in Pepper

**DOI:** 10.1155/2021/8105124

**Published:** 2021-01-25

**Authors:** Zhongrong Zhang, Ranran Zhu, Xuehua Ji, Hui Ji Li, Hui Lv, Hai Ying Zhang

**Affiliations:** College of Agriculture, Shihezi University, Xinjiang Production and Construction Corps Key Laboratory of Special Fruits and Vegetables Cultivation Physiology and Germplasm Resources Utilization, Shihezi, Xinjiang 832000, China

## Abstract

HD-ZIP is a unique type of transcription factor in plants, which are closely linked to the regulation of plant growth and development, the response to abiotic stress, and disease resistance. However, there is little known about the HD-ZIP gene family of pepper. In this study, 40 HD-ZIP family members were analyzed in the pepper genome. The analysis indicated that the introns number of Ca-HD-ZIP varied from 1 to 17; the number of amino acids was between 119 and 841; the theoretical isoelectric point was between 4.54 and 9.85; the molecular weight was between 14.04 and 92.56; most of them were unstable proteins. The phylogenetic tree divided *CaHD-ZIP* into 4 subfamilies; 40 *CaHD-ZIP* genes were located on different chromosomes, and all of them contained the motif 1; two pairs of *CaHD-ZIP* parallel genes of six paralogism genes were fragment duplications which occurred in 58.28~88.24 million years ago. There were multiple pressure-related action elements upstream of the start codon of the *HD-Z-IP* family. Protein interaction network proved to be coexpression phenomenon between *ATML1* (*CaH-DZ22, CaHDZ32*) and *At4g048909* (*CaHDZ12*, *CaHDZ31*), and three regions of them were highly homology. The expression level of *CaHD-ZIP* gene was different with tissues and developmental stages, which suggested that CaHD-ZIP may be involved in biological functions during pepper progress. In addition, Pepper HD-ZIP I and II genes played a major role in salt stress. *CaHDZ03*, *CaHDZ 10*, *CaHDZ17*, *CaHDZ25*, *CaHDZ34*, and *CaHDZ35* were significantly induced in response to salt stress. Notably, the expression of *CaHDZ07*, *CaHDZ17*, *CaHDZ26*, and *CaHDZ30*, homologs of Arabidopsis *AtHB12* and *AtHB7* genes, was significantly upregulated by salt stresses. *CaHDZ03* possesses two closely linked ABA action elements, and its expression level increased significantly at 4 h under salt stress. qRT-P-CR and transcription analysis showed that the expression of *CaHDZ03* and CaHDZ10 was upregulated under short-term salt stress, but *CaHDZ10* was downregulated with long-term salt stress, which provided a theoretical basis for research the function of *Ca-HDZIP* in response to abiotic stress.

## 1. Introduction

Plant transcription factors can be divided into 58 families according to the conserved domain and function [1]. Homeobox (HB) protein is a kind of transcription factor closely related to biological growth and development [2]. In 1983, Garber et al. [3] discovers the HB in Drosophila, and then, it is found in invertebrates, vertebrates, fungi, and other high plants. In 1991, Vollbrecht et al. [4] clones the HB gene with the name of Knotted-1 (Kn-1) in maize, and then, the HB gene is cloned in various plants. According to the location, differences, and homology of HD (homeodomain) sequences, plant HB proteins can be subdivided into six major categories: PHD-Finger, Bell, HD-ZIP, WOX, ZF-HD, and KNOX [5]. HD-ZIP transcription factor is a unique transcription factor in plants and plays roles in the growth, development, disease resistance, and abiotic stress [6]. These functions of HB have been reported in mass [7], ferns [8], monocotyledons [9], and dicotyledons [10, 11]. The HD-ZIP transcription factor is mainly composed of two conserved domains, HD (homeodomain) domain and a closely linked LZ (leucine zipper) domain. HD is linked to the specific binding of DNA, and Zip is related to heterodimerization [12]. According to structure and function, HD-ZIP proteins can be divided into four subfamilies: HD-ZIP I, HD-ZIP II, HD-ZIP III, and HD-ZIP IV [13].

HD-ZIP I protein is considered to be involved in the control of plant growth and development and the response to abiotic stress [14]. It is reported that there was a response of HD-ZIP I to acetic acid (ABA) at the transcript level under stress in *Arabidopsis* [15]. Under NaCl stress, the expressions of *Gmhdz51* and *Gmhdz 83* of cotton are substantially upregulated at 12 h after treatment [16]. In cotton, *GhHB1* (belongs to HD-ZIP I subfamily) may be involved in salt stress and ABA treatment, and the expression of *Gh-HB1* increases significantly in early stage of roots, and then decreases sharply, which suggests that HD-ZIP I played an important role in the early development of root [17]. In *Arabidopsis*, *AtHB1* works at a downstream location, and *AtPIF1* promotes hypocotyl elongation, particularly in response to short-day photoperiods [18], and mediates the apoptosis of leaf cell [19].

HD-ZIP II subfamily can induce shade avoidance reactions in plants through light signal transduction [20]. For example, when the seeds are stimulated with far-red light in the late germination period, they induce shade avoidance responses [21]. La Rota et al. [22] find that HD-ZIP III is involved in the *Arabidopsis* sepal development. HD-ZIP is also responsible for regulating plant cell differentiation and participating in the development of apical meristems, embryos, and vascular systems [15]. There are five family members of *Arabidopsis* HD-ZIP [13], due to differences in domains and expression patterns, each member plays different role [23–25]. The *PCN* gene in poplar belongs to the HD-ZIP subfamily and has a role in regulating xylem cell differentiation [26]. Transgenic poplars overexpressing *PCN* exhibited a slow deformation of wood and phloem and upregulation of endogenous hormone expression [28]. HD-ZIP IV subfamily is mainly involved in epithelial cell differentiation and root development [29]. In *Arabidopsis thaliana*, *PDF2*, *ATML1*, and *ATHB10* are HD-ZIPIV proteins, which have a regulatory effect on the specific expression of outer cortical cells [30], and the double mutants of *PDF2* and *ATML1* exhibit epidermal deletion [31]. The *OCL1* gene is a member of the maize HD-ZIP subfamily, and the N-terminal amino acid of its START domain plays a decisive role in the activity of this gene; overexpression can lead to delaying in flowering [32].

Pepper is a major vegetable, which is widely cultivated in the world. It is both fresh food, and processing raw material for seasoning, medicine, and cosmetics. It has very significant economic value [33]. The pepper genome has been the sequence in 2014 and can be used for gene prediction and annotation and public use [34, 35]. Several transcription factor families such as DOF, WRKY, AP2/ERF, and NAC in pepper have been studied [36–39]. HD-ZIP family genes have been reported in *Arabidopsis*, rice, poplar, corn, and other plants [40, 41], but so far, there is no systematic study of peppers HD-ZIP family. Our research analyzed the bioinformatic characteristics of the HD-ZIP gene family of pepper and supported a theoretical basis for studying the function of this gene in response to salt stress.

## 2. Materials and Methods

### 2.1. Genome-Wide Identification of HD-ZIP Family Genes in Pepper

We downloaded the pepper HD-ZIP protein sequence (PF00046 and PF02183) from the Pfam database (http://pfam.sanger.ac.uk/) and applied BLAST alignment to the pepper genome database PGP (http://peppergenome.snu.ac.kr/, the protein sequences annotated in CM334 and Zunla-1), the default parameters (Limit Expect Value 1e-5) output data, and the pepper CM334 was identified using the HMMER3.1 software (http://hmmer.org/). For all *CaHD-ZIP* proteins in the genome, the default output *E* value is <1 × 10 − 5, and the *CaHD-ZIP* sequence in the plant transcription factor database is combined to accurately obtain the target sequence [42]. With the help of NCBI-CDD (https://www.ncbi.nlm.nih.gov/ Structure/cdd/wrpsb.cgi) and SMART (http://smart.emblheidelberg.de/smart/save_user_preferences.pl) tools, protein domain identification was performed, and genes without the HD-ZIP main domain were deleted [43]. With the ExPASy-ProtParam tool (https://web.expasy.org/protparam/), the isoelectric point (pI) and molecular weight (MW) of the target sequence are predicted [44]. The online software Gene Structure Display Server (http://gsds.cbi.pku.edu.cn/) was used to predict the exon/intron structure of *CaHD-ZIP* [45].

### 2.2. Phylogenetic Analysis

To investigate the phylogenetic relationships of the HD-ZIPs among *C. annuum L*, *O. sativa*, and *A. thaliana*, multiple HD-ZIP protein sequences were aligned, and an unrooted phylogenetic tree was constructed in MEGA 5.05 [46]. The phylogenetic tree was constructed using the neighbor-joining (NJ) method. In the phylogenetic tree, group pattern was evaluated with bootstraps (1000 replicates).

### 2.3. Conserved Motif Analysis

The MEME software (http://meme-suite.org/tools/meme) was used to identify conserved motifs in *CaHD-ZIP*, the maximum number of motifs was 25, and other variables were the default values.

### 2.4. Chromosomal Location and Gene Duplication

The protein-coding sequence of *CaHD-ZIP* was mapped to the pepper genome database using BLASTn, and the gene was displayed on the chromosome by TBtools [47].

Plant gene duplication database (Plant Genome Duplication Database PGDD http://chibba.agtec.uga.edu/duplication/index/locus) was used to identify the gene duplication of pepper HD-ZIP gene [48]. Ka, Ks, and Ka/Ks were estimated using DnaSPV5 [49]. The Ks value of each pair is used to estimate the replication time by the following formula: replication time = Ks/2*λ*, where *λ* = 6.1 × 10^−9^.

### 2.5. Promoter Element Analysis

The upstream region (2 kb) of the *CaHD-ZIP* gene is derived from PGD database (http://peppergenome.snu.ac.kr). The promoter elements of *CaHD-ZIP* family members are predicted by the PLACE [50] (http://www.dna.affrc.go.jp/PLACE), expressed through TBtools.

### 2.6. Prediction of Protein-Protein Interaction Network

Based on the phylogenetic relationship between the CaHD-ZIP protein and the *Arabidopsis* HD-ZIP protein, 32 *Arabidopsis* HD-ZIP proteins which represent the 40 pepper HD-ZIP proteins are uploaded to the string website [51] (https://string-db.org/) to predict protein interactions. The online program run with default parameters.

### 2.7. Transcriptome Analysis of CaHD-ZIP in Different Tissues

Based on the CM334 RNA-seq [34], the expression patterns of *CaHD-ZIP* in different stages and tissues were analyzed; the tissues include root, stem, leaf, pericarp (PC), and placenta (PL) at 6, 16, and 25 days postanthesis (DPA), PC and PL at mature green (MG) and at breaker (B) stages, and PC and PL at 5 and 10 days postbreaker (B5 and B10, respectively).

### 2.8. Plant Cultivation and NaCl Stress

Healthy pepper seeds (*Capsicum annuum* L. var. conoides (Mill) Irish) were soaked at room temperature for 6-8 h; then, the seeds were placed in a germination box and covered with wet gauze at 28 ± 2°C. The germinated seeds were sown into a pot with substrate (Vpeat : Vvermiculite = 2 : 1). When the sixth leaves appeared, the seedlings were transferred to barral with 8 L distilled water. Each barrel had four peppers. After a week, the seedlings were treated with 100 mM NaCl solution, and those cultivated with distilled water were control. The pH was adjusted to 7.0 using H2SO4 or NaOH. The solution was replaced every three days. After the treatment of 4 h and 58 h, the young leaves were sampled and stored at -80°C until Illumina sequencing. Total RNA was extracted from the pepper leaf with RNA out 1.0 (Tianenze, Beijing, China), and RNA quality was examined by NanoDrop (Thermo Fisher Scientific, Inc.) and Agilent 2100 Bioanalyzer (Agilent Technologies, Santa Clara, CA, USA). cDNA library construction and Illumina RNA-Seq (HiSeq TM-2500) were carried out by Novo Gene Company (Beijing, China) [52].

### 2.9. Total RNA Extraction and cDNA Synthesis

After the stress of 4 h and 58 h, total RNA was extracted from pepper leaves with an RNA concentration of 1.0 (Tianenze, Beijing, China). Each group has three biological replicates. Primers were designed according to Premier 6.0 (S5). cDNA was synthesized using the reverse transcriptase MMLV kit (China ABM). Real-time quantification was performed using the CFX Manager (Bio-Rad, USA) and the SYBR Green Real-Time PCR Master Mix (Abm, Canada). The protocol of real-time PCR was as follows: predenaturation at 95°C for 10 minutes, then 40 rounds of amplification at 40°C, 15 s denaturation at 95°C, and annealed at 30°C for 30 s, extended to 72°C and read the plate to record fluorescence data at 65°C. Melting curves were performed at 65°C to 95°C to check the specificity for the amplified products. Each reaction was repeated three times. Pepper ACTIN1 was used as an internal control.

## 3. Results

### 3.1. Identification and Structure Analysis of the CaHD-ZIP Gene Family in Pepper

40 *CaHD-ZIP* target sequences were obtained, which were named with CaHD-ZIP01 to CaHD-ZI-P40 (Table 1). It could be seen from Table 1 that the number of amino acids in each CaHD-ZIP sequence was between 119 and 841; the theoretical isoelectric point was between 4.54 and 9.85; the molecular weight was between 14.04 and 92.56 kD; the instability coefficient results showed that except *CaHDZ29* and *CaHDZ32*, the other 38 *CaHD-ZIP* family members were unstable proteins (Table 1). The core domain analysis showed that the CaHD-ZIPI subfamily had two domains, HD and LZ. Report to the CaHD-ZIPI, CaHD-ZIPII had an additional N-term. For CaHD-ZIP III and CaHD-ZIP IV subfamilies, in addition to the HD and LZ domains, they had a START domain and a MEKHLA domain (S1A). Analysis of the predicted introns and exons of the pepper HD-ZIP gene revealed that 5 genes (*CaHDZ 03*, *CaHDZ17*, *CaHDZ23*, *CaHDZP35*, and *CaHDZ38*) had no introns in the pepper genome. Most *CaHD-ZIP* genes contained 3-18 exon in the coding DNA sequence. 216 introns had 0 phases, and 1 had 2 phases (S1 B).

### 3.2. Phylogenetic Analysis of CaHD-ZIP Genes

**Figure 1:**
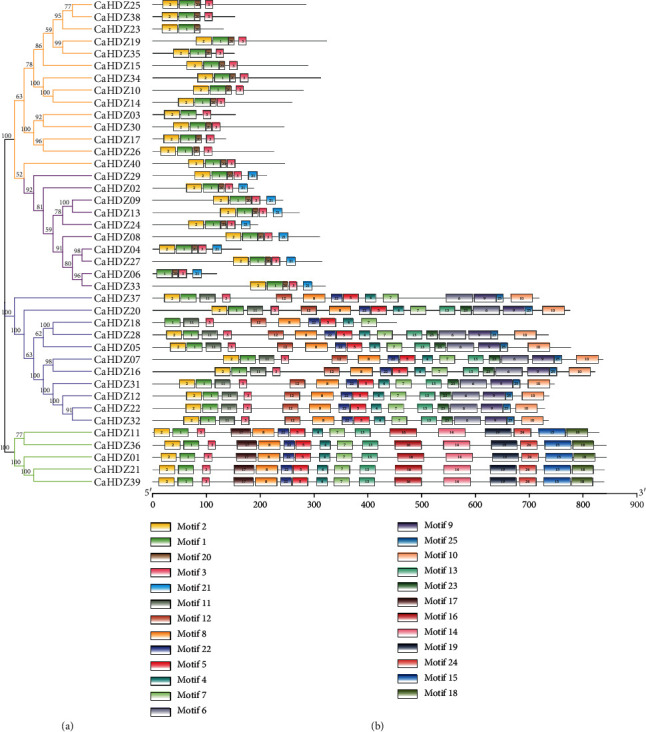
Unrooted neighbor-joining phylogenetic tree and conserved motif analysis of *CaHD-ZIP* proteins. (a) The phylogenetic tree was generated based on the protein sequences of *CaHD-ZIP* proteins. (b) Conserved motif analysis of *CaHD-ZIP* proteins. Different color boxes represent different types of motifs.

In order to examine the phylogenetic relationship between the 32 HD-ZIP transcription factors in pepper and the known members of other plants, we created a rootless development tree between *Arabidopsis*, rice, and pepper and implemented it in the MEGA 6 software. The phylogenetic tree implied that there are four groups of HD-ZIP, which is similar to previous studies on sesame, poplar, and corn [40, 41, 53]. A number of HD-ZIP I to HD-ZIP IV members of pepper are 14, 10, 5, and 11. The tree also showed that most *CaHD-ZIP* proteins move closer to members of Arabidopsis thaliana than members from rice (Figure 1). For example, in the third group, *CaHD-ZIP 11* was clustered with *AtPHV* and *AtPHB*, while *Oshox 32* and *Oshox 33* in the third group are clustered in a single clade. These results offered an important basis for the prediction of the function of pepper HD-ZIP protein.

### 3.3. Analysis of Conserved Motifs of the CaHD-ZIP Gene Family

The conserved motif of *CaHD-ZIP* protein was further analyzed using the meme software. The software detected a total of 25 motifs in the 40 *CaHD-ZIP* proteins, which were designated 1 to 25 (Figure 1 and S2). As expected, all identified *CaHD-ZIP* proteins (except *CaHDZ06, 18*) contained the LZ domain (motif 3) and the HD domain (motifs 1 and 2). A START field (topic 8) was found in the members of the third and fourth groups but was not found in the first and second groups. Among the third category, Motif15 and Motif18 were found to correspond to the Mekhla domains e. In addition to these, new functional motifs, some domains with unknown functions were also discovered, for example, Motif 6, 9, 10, 11, 12, 23, and 25 (only detected members of the fourth group) and Motif 14, 17, and 19 (only found in members of the third group). The results also indicated that members of the same group of *CaHD-ZIP* usually had similar motifs and therefore may have similar function.

### 3.4. Chromosomal Locations and Syntenic Analysis

**Figure 2:**
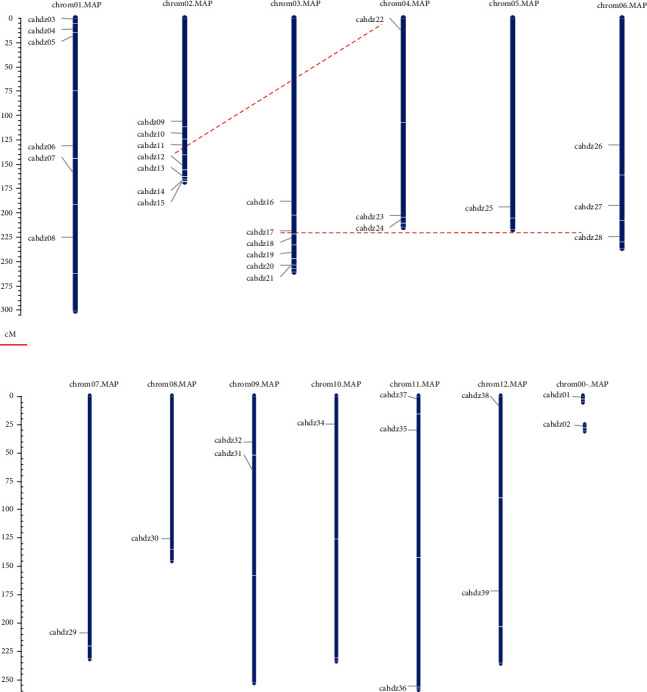
Chromosomal locations and duplication events of *CaHD-ZIP* genes of pepper. Locations of *CaHD-ZIP* were based on physical locations. The numbers on the top represent the chromosome number. The chromosome 00 (Chr00) means two different scaffolds containing unassigned *CaHD-ZIP* to any of the 12 pepper chromosomes. Red lines indicate 2 pairs of paralogism genome duplication events.

40 *CaHD-ZIP* genes were located on twelve chromosomes, 7 *CaHD-ZIP* genes located on chromosome 02 (*CaHDZ09*, *10*, *11*, *12*, *13*, 14 and *CaHDZ15*), 6 *CaHD-ZIP* genes were located on the long arms of chromosome 01 and 03, chromosomes 4, 6, 11, and 12 included 3 genes, respectively, and chromosomes 09 and 10 had two genes, respectively, each of chromosome of 5, 7, and 8 had one *CaHD-ZIP* gene, and chromosome 0 had two genes of *CaHDZ01* and *CaHDZ02* (Figure 2).

Analysis of HD-ZIP genes duplication of pepper showed that two pairs of paralogism genes (*CaH-DZ18* and *CaHDZ28* and *CaHDZ12* and *CaHDZ22*) were fragment replication. The nonsynonymous substitution rate (Ka), the synonymous substitution rate (Ks), and the Ka/Ks ratio were shown in S3. It was reported that *CaHD-ZIP*'s two-segment replication happened between 58.28 million and 88.24 million years ago. The Ka/Ks values of the two replication pairs were lower than 0.3, which indicated that there were no significant functional differences between these *CaHD-ZIP* genes after the replication event.

### 3.5. Promoter Analysis of the CaHD-ZIP Proteins

**Figure 3:**
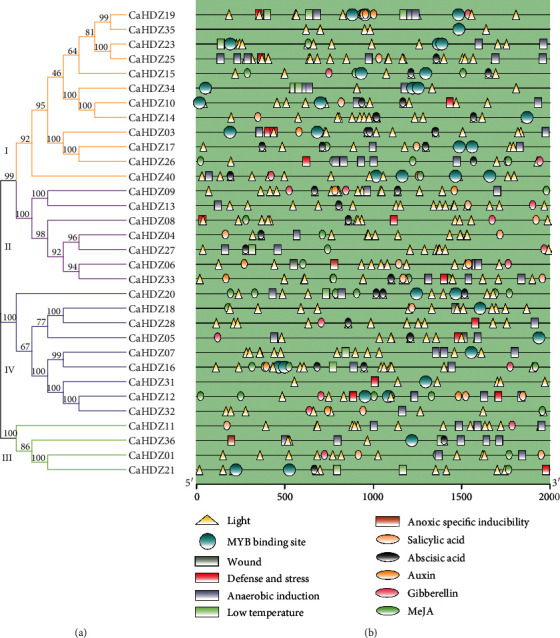
Cis analysis of *CaHD-ZIP* promoter related to stress response. Different cis-elements with the same or similar functions are shown in the same shape and color.

To further explore the possible regulation mechanism of *CaHD-ZIP* under various pressure, the case element in the promoter sequence of *CaHD-ZIP* gene was studied. The cis-elements are divided into four main subgroups: stress-response, hormone responsiveness, photosensitivity, and MYB binding sites [54] (Figure 3, S6). In our study of 32 *CaHD-ZIP* genes, 19 of them possessed acetic acid response elements and 13 possessed low-temperature response originals. In addition, we got a large number of light-responsive cis-elements in *CaHD-ZIP*, especially in *CaHDZ13* gene, 19 MYB binding sites, and 12 defense stress-response elements. It was useful to noting that the wound response element was only found in three members (*CaHDZ05*, *CaHD06*, and *CaHD18*), and hypoxia-specific induction was only found in two members (*CaHDZ06* and *CaHDZ36*). *CaHDZ03* has two closely connected defensive stress response and ABA action elements.

### 3.6. Prediction of Protein-Protein Interaction Network

**Figure 4:**
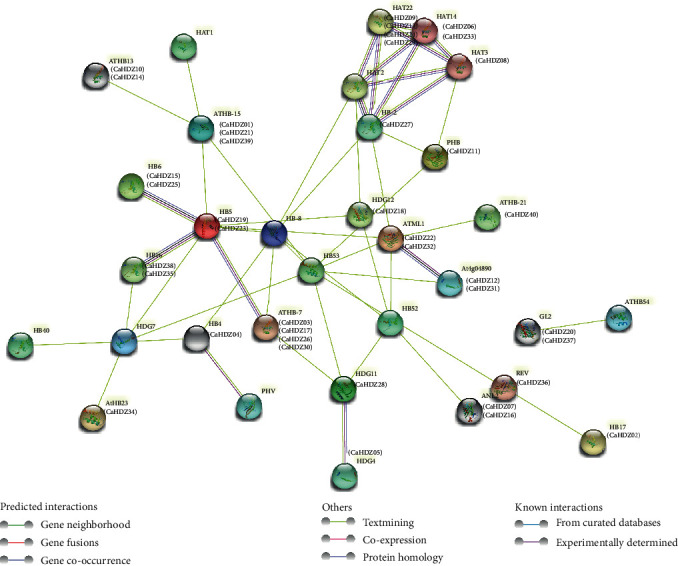
Predicted protein-protein interaction network of *CaHD-ZIP* proteins. The colors of the line indicate different data sources.

In order to further understand the interaction of pepper HD-ZIP proteins, an interactive network based on *Arabidopsis* orthodoxy was established using STRING. The results showed that there was just one pair of *ATML1* (*CaHDZ22*, *32*) and *At4g048909* (*CaHDZ12*, *31*). There was a coexpression phenomenon, and the protein homology between the two was high. It has been confirmed by the protein two-hybrid test on *Arabidopsis* [55]. Three high homology regions were verified, one of them was located on *HB-2* (*CaHDZ27*), and it had high homology to *HAT22* (*CaHDZ9*, *13*, *24*, *29*), *HAT14* (*CaHDZ06*, *33*), and *HAT3* (*CaHDZ08*). The second region was centered on *HB5* (CaHDZ19, 23), and it had high homology with *HB6* (*CaHDZ15*, *25*), *HB16* (*CaHDZ35*, *38*), and *ATHB-7* (*CaHDZ03*, *17*, *26*, *30*). The last area was a high correlation with *HDG11* (*CaHDZ28*) and *HDG4* (*CaHDZ05*). The relationship of other HD-ZIPs had yet to be explored (Figure 4).

### 3.7. Expression Analysis of CaHD-ZIP Genes in Different Tissues and Development Stages

**Figure 5:**
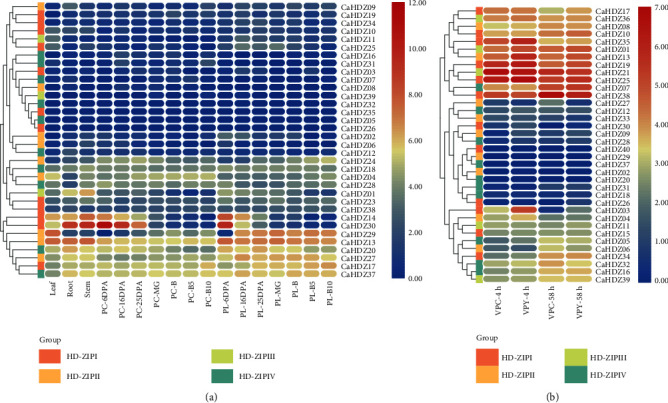
(a) Pepper HD-ZIPgenes expression of different tissues and developmental stages. Raw data were taken from RNA-seq data of CM334. The tissues included root, stem, leaf, pericarp (PC), and placenta (PL) at 6, 16, and 25 days postanthesis (DPA), PC and PL at mature green (MG) and at breaker (B) stages, PC and PL at 5 (B5) and 10 (B10) days after postbreaker, respectively. (b) The heat map of *CaHD-ZIP* genes under NaCl treatment (VPC represents CK, VPY represents NaCl stress). Blue and red colors represent relatively low and high expression (log 2 RPKM value).

To study the role of *CaHD-ZIP* in pepper growth and development, we cited publicly available RNA-seq data from 5 tissues (root, stem, leaf, peel, and placenta) to generate *CaHD-ZIPs* heat map of transcription patterns, which included seven developmental stages of the peel and placenta (Figure 5(a), S5). The expression profile of each *CaHD-ZIP* gene revealed various patterns in different organs and stages. *CaHDZ 04*, *13*, *17*, *18*, *24*, *28*, *37* had higher expression levels in all tissues; *CaHDZ 01*, *14*, *30* had higher expression levels in roots, stems, peels, and placenta; *CaHDZ11*, *25* had higher expression in leaf and placentas. The expression level of *CaHDZ35*, *26*, *05*, *32* in each tissue was relatively low. The expression levels varied with the developmental stages significantly, for example, *CaHDZ14* was highly expressed in PC-6DPA, PL-6DPA, and PL-16DPA, but hardly expressed in PC-B10 and PL-B5; the expression of *CaHDZ30* in PC-6DPA was the highest. The expression of *CaHDZ29* in PL (including PL-16DPA, PL-25DPA, PL-MG, PL-B, PL-B5, and PL-B10) was higher than that of other periods. The expression level of *CaHDZ27* was higher throughout the PL period. The expression level of *CaHDZ20* in PC-B, PC-B5, and PC-B10 was relatively low PL period. These results suggested that *CaHD-ZIP* may be involved in biological functions of pepper development.

### 3.8. CaHD-ZIP Expression Profile under Salt Stress

The expression pattern of *CaHD-ZIP* transcription factor under salt stress was obtained from the transcription data (Figure 5(b), S4). It was noted that *CaHDZ 01*, *CaHDZ04*, *CaHDZ 09*, *CaHDZ32*, *CaH-DZ13*, *CaHDZ19*, and *CaHDZ35* had higher expression levels after 4 h of salt stress, and *CaHDZ 03*, *CaH-DZ11*, and *CaHDZ17* had higher expression after 58 h of salt stress. Expression levels of *CaHDZ 06* and *Ca-HDZ27* were lower than those of the control after 4 h and 58 h of salt stress. It was useful to note that the expression level of *CaHDZ03* increased significantly after 4 h of salt stress.

### 3.9. Expression Analysis of CaHD-ZIP Genes in Response to Salt Stresses

**Figure 6:**
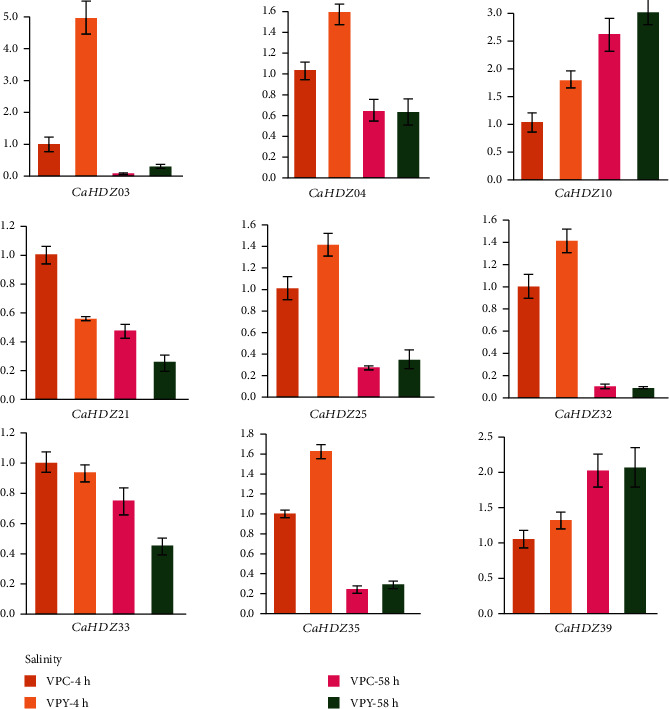
The heat map of the *CaHD-ZIP* genes under NaCl treatment (VPC represents CK, VPY represents NaCl stress). Expression patterns of 9 *CaHDZ* genes under salt stress. Salt stress (100 mM NaCl) was carried out on pepper seedlings at the six-leaf stage. The relative expression level of *CaHDZ* gene was analyzed by qRT-PCR, using sesame VPC4h gene as the internal control.

Based on the differential expression data of the transcription, 9 genes were selected in different subfamilies, and qRT-PCR was used to find out the role of the gene under salt stress (Figure 6). The expression levels of *CaHDZ03*, *CaHDZ04*, *CaHDZ25*, *CaHDZ32*, *CaHDZ35*, and *CaHDZ39* were higher than that of the control after 4 h salt stress; the expression levels of *CaHDZ03*, *CaHDZ04*, *CaHDZ10*, *C-aHDZ21*, and *CaHDZ33* decreased after 4 h of salt stress. The expression of the *CaHDZ21*, *CaHDZ33*, and *CaHDZ32* genes decreased after 58 h of salt stress. *CaHDZ03* and *CaHDZ10* can be induced to increase the expression level under short-term salt stress and decreased expression level under long-term salt stress. These results suggested that these genes may play a significant role in peppers response to salt stress.

## 4. Discussion

HD-ZIP, as a plant-specific transcription factor, is closely linked to abiotic stress. HD-ZIP transcription factors are widely disseminated in different plants. HD-ZIP genes are highly conserved, but their proteins are different. This study got 40 HD-ZIP transcription factors; those factors contain a highly conserved domain, but most members are unstable proteins. We analyzed 217 introns, 216 of them have phase 0, only one of them has phase 2, and all the introns were available on both sides of the exon. This phase was called symmetrical exons. Based on the early intron hypothesis [56], excessive phase 0 introns and symmetrical exons can effectively promote exon shuffling by avoiding the interruption of the open reading frame, which could accelerate the recombination and exchange of protein domains [57]. There were a large number of phase 0 introns and symmetric exons of *CaHD-ZIP* genes in our study. It was corresponding with the statement and indicates that exon shuffling may be playing an important role in the evolution.

**Figure 7:**
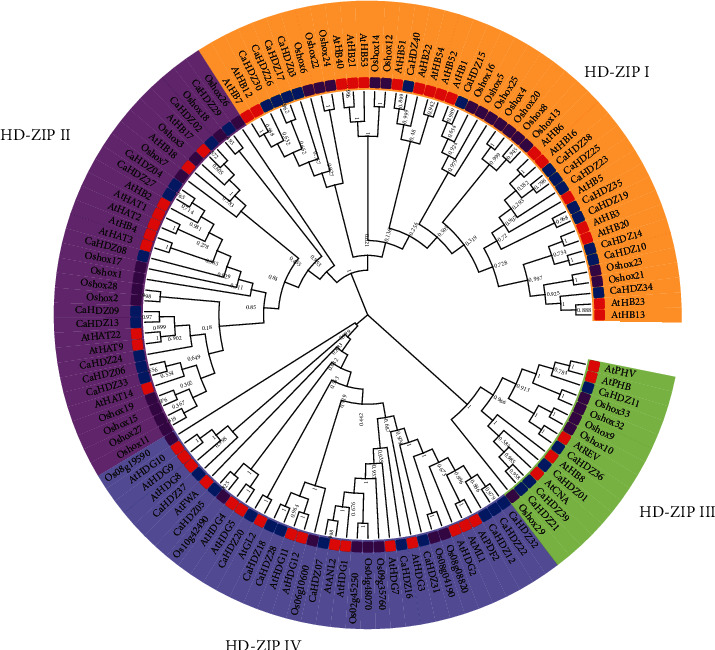
Phylogenetic trees of 132 HD-ZIP proteins in pepper (blue square), *Arabidopsis* (red square), and rice (purple square). The HD-ZIP gene is divided into four subfamilies (groups I, II, III, and IV, with yellow, purple, green, and blue branches, respectively).

The phylogenetic tree was constructed using HDZ proteins of pepper, rice, and *Arabidopsis*. HDZ proteins of pepper can be divided into four categories (HD-ZIP I-IV), and the number of HD-ZIP I, II, III, and IV are 14, 10, and 5, respectively, 11, and 17, 10, 5, and 16 in *Arabidopsis* [58]; 12, 9, 4, and 7 in physic nut [59]; 11, 7, 5, and 8 in grape [60]; 16, 10, 9, and 10 in sesame [53]. These results suggested that the HD-ZIPI protein was the most abundant type of pepper HD-ZIP transcription factor, and the amount of HD-ZIPIII protein in pepper was the same as that of most plants. In addition, the phylogenetic tree also showed that most CaHDZ proteins were closer to members of *Arabidopsis thaliana* than members from rice [59] (Figure 7. Motif analysis also demonstrated that *CaHDZ* protein motifs distributed differently with subfamilies, and genes of the same family had similar structure and function (Figure 1, S2), which provided a powerful guarantee for the evolution of pepper. Gene duplication is the highest evolutionary mechanism that helps plants adapt to various environmental stresses [15]. Compared with 41 pairs of soybean [16] and 10 pairs of cassava [61] paralogism HD-ZIP genes, there were only two pairs of pepper, *CaHZ18* and *CaHDZ28* and *CaHDZ12* and *CaHDZ22*; those genes distributed on different chromosomes. Paralogous genes originated from the partial duplication of *CaHD-ZIP* between chromosomes. Replication of two fragments occurred between 5828 and 88.24 million years ago. The genomes of most polyploid plants contain a large number of repetitive chromosome segments, so the probability of partial repetitions is significantly higher than that of tandem repeats and translocations [62]. It speculated that the evolution of the HD-ZIP family of pepper was slow.

More and more evidence shows that the HD-ZIP gene is associated with plant growth and development [16, 40, 53]. For example, *AtHB2* regulates the shade response of red light/far red light, which is linked to the formation of lateral roots. In sesame, HD-ZIP I, II, and III are widely expressed in all tissues [53], and similar phenomena had been found in pepper, but the number of *CaHDZ* genes expressed was less than that of sesame. In addition, we also found that the *CaHD-ZIP*IV gene in pepper showed a clear tissue-specific expression pattern (Figure 5). For instance, *CaHDZ28* was hardly expressed in roots but was expressed in other tissues; the expression of *CaHDZ12* was opposite to that of *CaHDZ28*. HD-ZIP IV gene is highly expressed in young leaves and flowers of tomato [63]. These results indicated that different subgroups of HD-ZIP genes have distinct functions. Members of the *CaHD-ZIP* family have multiple pressure-related action elements upstream of the start codon. These case elements are divided into four main subgroups: stress-response, hormone responsiveness, photosensitivity, and MYB binding site [54]. ABA response element, low-temperature response element (LTRE), and dehydration response element (Dre) are the main transcription factors regulating ABA signal transduction and participate in salt and drought stress [64]. *CaHD-ZIP* gene contained ABA signal transduction, and the greater the number of related action elements, the higher the gene expression level under salt stress. A lot of evidence shows that HD-ZIP I protein is involved in developmental reprogramming, c and coping with environmental pressure [65]. For example, *AtHB7* and *AtHB12* can act as negative feedback on the effect of ABA signal in plants under water shortage [65, 66]. Some of the HD-ZIP I genes in pepper, such as *CaHDZ03*, *10*, *17*, *25*, *34*, and *35*, were significantly induced in response to salt stress. Notably, the expression of *CaHDZ07*, *17*, *26*, and *30*, homologs of Arabidopsis *AtHB12* and *AtHB7* genes, was markedly upregulated by salinity stresses, indicating that these genes may regulate drought and salt tolerance through an ABA-dependent pathway. In particular, *CaHDZ03* possessed two closely linked defense stress responses and ABA action elements, and its expression level increased significantly at 4 h under salt stress. The correlation analysis of qRT-PCR (Figure 6) and transcription data (S4) showed that the expression of *CaHDZ03* gene can be upregulated under short-term salt stress. In addition, we found that the defense stress-response may also be related to those gene expressions which contain defense stress-response elements. *CaHDZ03*, *CaHDZ19*, *CaHDZ25*, and *CaHDZ26* had higher expressions than other genes under salt stress; *CaHDZ10* was increased under short-term salt stress but decreased under long-term salt stress.

## 5. Conclusion

In the present study, we identified 40 HD-ZIP genes in pepper, including four types, which were unevenly distributed on 12 chromosomes. Syntenic analysis showed that *CaHDZ18*, *CaHDZ28*, *CaHDZ12*, and *CaHDZ22* are fragment duplication. Two fragment duplications occurred at 58.28~88.24 million years ago. There were multiple upstream of the start codon of HD-ZIP family members. There was coexpression between *ATML1* (*CaHDZ22*, *CaHDZ32*) and *At4g048909* (*CaHDZ12*, *CaHDZ31*), and there were three regions with high homology of them. Expressions of the CaHD-ZIP gene ranged with plant tissue and developmental stage. The HD-ZIPI responded more significantly to salt stress than other subfamilies.

## Figures and Tables

**Table 1:** Characteristics of CaHD-ZIP genes from Zunla-1 genome and CM334 genome. AA: amino acid; pI: the theoretical isoelectric point of proteins; Mw: the theoretical molecular weight of proteins.

Gene name	Gene ID	Chromosome or scaffold	No. of AA	pI	MW (kD)	Instability index	NO. of introns
CaHDZ01	CA00g45190	scaffold1113	841	5.89	92.36	52.91	17
CaHDZ02	CA00g57460	scaffold1298	187	9.60	21.48	61.60	4
CaHDZ03	CA01g00300	1	153	9.68	17.64	62.20	0
CaHDZ04	CA01g05840	1	164	9.50	18.89	67.79	2
CaHDZ05	CA01g07870	1	775	5.30	86.33	57.69	9
CaHDZ06	CA01g17370	1	119	9.85	14.04	75.19	1
CaHDZ07	CA01g19070	1	835	5.86	91.07	50.23	8
CaHDZ08	CA01g27890	1	309	6.77	34.32	70.60	3
CaHDZ09	CA02g07270	2	241	9.03	27.00	48.52	2
CaHDZ10	CA02g08550	2	279	5.79	31.69	55.49	2
CaHDZ11	CA02g10530	2	828	5.93	91.56	47.30	17
CaHDZ12	CA02g18040	2	735	5.70	81.01	41.35	9
CaHDZ13	CA02g24650	2	272	8.40	30.28	49.85	2
CaHDZ14	CA02g28390	2	258	5.69	29.49	62.30	1
CaHDZ15	CA02g29480	2	287	4.82	32.42	74.16	3
CaHDZ16	CA03g16380	3	820	6.07	89.03	45.35	8
CaHDZ17	CA03g20530	3	135	9.81	16.29	55.67	0
CaHDZ18	CA03g23110	3	452	5.56	50.94	48.73	5
CaHDZ19	CA03g28010	3	322	5.03	37.08	52.26	2
CaHDZ20	CA03g34750	3	774	6.71	86.78	53.61	10
CaHDZ21	CA03g35060	3	838	6.06	92.05	47.62	17
CaHDZ22	CA04g03990	4	728	5.53	80.14	42.32	9
CaHDZ23	CA04g15670	4	131	8.56	15.48	55.19	0
CaHDZ24	CA04g16620	4	194	8.86	22.42	59.32	2
CaHDZ25	CA05g13390	5	284	4.54	32.23	50.11	1
CaHDZ26	CA06g08530	6	224	5.17	25.98	48.36	1
CaHDZ27	CA06g12600	6	313	8.13	35.61	59.61	3
CaHDZ28	CA06g20810	6	734	6.08	80.87	52.29	9
CaHDZ29	CA07g14020	7	211	9.35	24.26	37.29	2
CaHDZ30	CA08g08650	8	243	5.27	27.63	58.24	1
CaHDZ31	CA09g07360	9	745	5.48	82.68	41.08	9
CaHDZ32	CA10g05400	10	734	5.66	80.87	39.58	9
CaHDZ33	CA10g19210	10	321	8.88	36.14	58.46	3
CaHDZ34	CA11g05100	11	311	5.73	35.61	62.75	2
CaHDZ35	CA11g05650	11	151	6.33	17.80	46.65	0
CaHDZ36	CA11g18960	11	841	5.84	92.13	51.57	17
CaHDZ37	CA12g00830	12	716	5.90	81.57	45.82	8
CaHDZ38	CA12g03310	12	152	6.13	17.83	49.39	0
CaHDZ39	CA12g13110	12	837	6.04	92.56	47.05	17
CaHDZ40	Capana09g002322^∗^	9	245	7.77	28.43	54.10	2

## Data Availability

The attached table contains all the data used to fund the results of this study. Transcription data under salt stress has not been uploaded to the NCBI because this article has not been published, but S4 contains the data needed for this article. The qRT-PCR data used to support the findings of this study are included within the supplementary information file (Table S7).

## Supplementary Materials

Supplementary 1 Table S1: the main domain and gene structure information of pepper HD-ZIP protein sequence.

Supplementary 2 Table S2: conserved motifs of CaHD-ZIP family proteins, used to analyze the conserved sequences of pepper.

Supplementary 3 Table S3: the Ka/Ks ratios for CaHD-ZIPproteins which calculate using DnaSPV5.

Supplementary 4 Table S4: transcriptome data (including raw data for analysis of tissue and expression under salt stress).

Supplementary 5 Table S5: the specific primer for qRT-PCRof each CaHD-ZIP gene.

Supplementary 6 Table S6: pepper HD-ZIP gene promoter function element.

Supplementary 7 Table S7: correlation between HD-ZIP gene RNA-seq data and qRT-PCR of 9 pepper.
