# Supplementary material for: Genome-Wide Characterization and Expression Analysis of the HD-ZIP Gene Family in Response to Salt Stress in Pepper
Source: Int J Genomics. 2021 Jan 25;2021:8105124. doi: 10.1155/2021/8105124 (PMC7869415; doi:10.1155/2021/8105124)
Supplement: Supplementary 1 — Table S1: the main domain and gene structure information of pepper HD-ZIP protein sequence. [file 8105124.f1.docx]

| 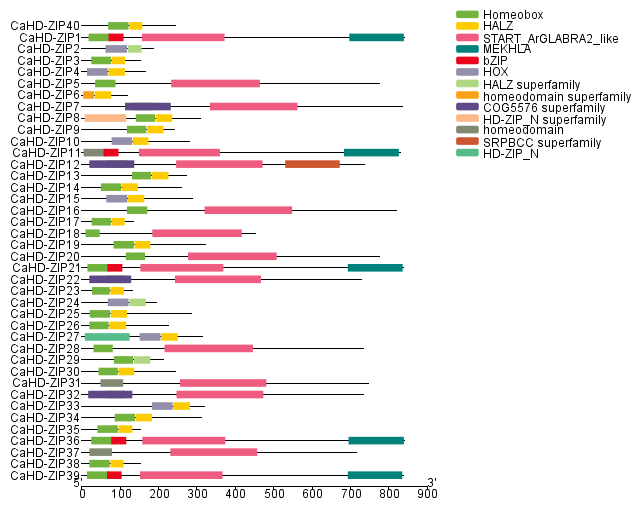  **S1A:**the main domain of pepper HD-ZIP protein sequence. | 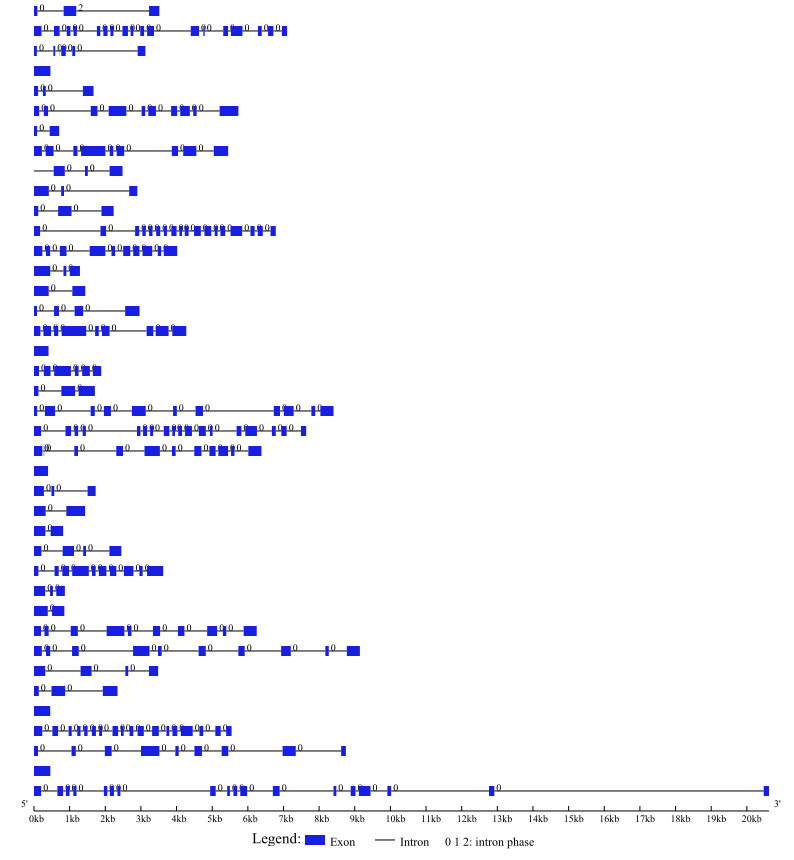  **S1B: the gene structure information of pepper HD-ZIP protein sequence.** |
| --- | --- |
