# Supplementary material for: Genome-Wide Characterization and Expression Analysis of the HD-ZIP Gene Family in Response to Salt Stress in Pepper
Source: Int J Genomics. 2021 Jan 25;2021:8105124. doi: 10.1155/2021/8105124 (PMC7869415; doi:10.1155/2021/8105124)
Supplement: Supplementary 2 — Table S2: conserved motifs of CaHD-ZIP family proteins, used to analyze the conserved sequences of pepper. [file 8105124.f2.docx]

Motif of the CaHD-ZIP family protein domain

| Motif | Width | Multilevel consensus sequence |
| --- | --- | --- |
| 1 | 29 | LARELGLQPRQVKVWFQNRRARWKTKQEE |
| 2 | 29 | EKKRRLTKEQVQALEKSFKECPKLDPKQK |
| 3 | 15 | LREENERLKKEVQEL |
| 4 | 20 | CRRLPSGCLIQELPNGYSKV |
| 5 | 29 | LVPTREFYFLRYCKQHEDGSWAVVDVSLD |
| 6 | 50 | WLPVSPKRVFDFLRDENSRSEWDILSNGGPVQEMAHIANGRDPGNCVSLL |
| 7 | 29 | VHEIYRPLVESGLAFGAKRWVATLQRQCE |
| 8 | 41 | IGAKSHGCTGEASRESGVVIMNPIRLVEILMDRNKWFEDFP |
| 9 | 41 | SQSNMLILQESCTDSTGSYVIYAPVDIAAMNVVLSGGDPDY |
| 10 | 29 | RAENDKLRAENIRYREALKNVTCPNCGGP |
| 11 | 29 | KSLIIELALAAMEELIRMAQTGEPLWIKS |
| 12 | 29 | SLLKLAZRMVRSFCAAVNASTDHGWTTLS |
| 13 | 34 | QIMQQGFACLQGGICLSSMGRPISYERAVAWKVL |
| 14 | 50 | LWHHSDAIICCSLKALPVFTFANQAGLDMLETTLVALQDISLEKIFDEHG |
| 15 | 50 | IMPRDMFLLQLCSGMDENAVGTCAELVFAPIDASFADDAPLLPSGFRIIP |
| 16 | 41 | SPAGLLSIAEETLTEFLSKATGTAVEWVQMPGMKPGPDSIG |
| 17 | 50 | LCAKASMLLQNVPPAILLRFLREHRSEWADNNIDAYSAAAVKAGPCSJPG |
| 18 | 33 | LLTVAFQILVESHPTAKLSLMSVATVNSLISCT |
| 19 | 48 | QRVALAJSPSRJGSLGGLRLPPGTPEAHTLARWICQSYRYFLGVELLK |
| 20 | 11 | DYEILKRCYDS |
| 21 | 16 | LPATTLTMCPSCERVA |
| 22 | 21 | GVAGNYNGTJZLMYAELQVPT |
| 23 | 21 | MTRKSVDDPGRPPGIVLSAAT |
| 24 | 14 | VALLPSGFAILPDG |
| 25 | 10 | TWVEHVEVDE |

Note: The serial number of the phantom is the same as the figure in the text
