# Supplementary material for: Genome-Wide Characterization and Expression Analysis of the HD-ZIP Gene Family in Response to Salt Stress in Pepper
Source: Int J Genomics. 2021 Jan 25;2021:8105124. doi: 10.1155/2021/8105124 (PMC7869415; doi:10.1155/2021/8105124)
Supplement: Supplementary 3 — Table S3: the Ka/Ks ratios for CaHD-ZIPproteins which calculate using DnaSPV5. [file 8105124.f3.docx]

S 3 Syntenic Analysis of CaHD-ZIP family proteins

| Gene1 | Gene2 | Ka | AKs | Ka/Ks BDate(Mya) | |
| --- | --- | --- | --- | --- | --- |
| CaHDZ18 | CaHDZ28 | 0.12 | 0.71 | 0.17 | 58.28 |
| CaHDZ12 | CaHDZ22 | 0.13 | 1.07 | 0.12 | 88.24 |

A： DnaSPV5 is used to calculate Ka and Ks.

B：The replication time is estimated according to the formula: T-Ks/2- and t-6.1 x 10-9.

Mya：million years ago。
