# Supplementary material for: Genome-Wide Characterization and Expression Analysis of the HD-ZIP Gene Family in Response to Salt Stress in Pepper
Source: Int J Genomics. 2021 Jan 25;2021:8105124. doi: 10.1155/2021/8105124 (PMC7869415; doi:10.1155/2021/8105124)
Supplement: Supplementary 4 — Table S4: transcriptome data (including raw data for analysis of tissue and expression under salt stress). [file 8105124.f4.docx]

| **Table S4A: the transcriptome data (including raw data for analysis of tissue** | | | | | | | | | | | | | | | | | |
| --- | --- | --- | --- | --- | --- | --- | --- | --- | --- | --- | --- | --- | --- | --- | --- | --- | --- |
| gene ID | Leaf | Root | Stem | PC-6DPA | PC-16DPA | PC-25DPA | PC-MG | PC-B | PC-B5 | PC-B10 | PL-6DPA | PL-16DPA | PL-25DPA | PL-MG | PL-B | PL-B5 | PL-B10 |
| CaHDZ01 | 10.234 | 40.674 | 78.147 | 6.909 | 6.066 | 8.494 | 3.833 | 1.104 | 0.343 | 1.19 | 22.903 | 16.307 | 12.566 | 10.543 | 6.853 | 2.144 | 1.694 |
| CaHDZ02 | 0 | 0.935 | 1.989 | 1.786 | 0.757 | 0.989 | 0.307 | 0.438 | 0.14 | 0.874 | 7.746 | 5.76 | 1.691 | 2.108 | 2.802 | 0 | 0.418 |
| CaHDZ03 | 0.393 | 0.24 | 0.555 | 0 | 0.67 | 1.198 | 0.959 | 1.249 | 0 | 0 | 0.302 | 1.977 | 1.134 | 0 | 0 | 1.396 | 0 |
| CaHDZ04 | 41.282 | 3.562 | 17.206 | 11.76 | 14.127 | 26.084 | 29.161 | 29.96 | 32.404 | 33.215 | 8.419 | 8.628 | 7.673 | 8.307 | 8.401 | 13.292 | 18.633 |
| CaHDZ05 | 0 | 0 | 0 | 0 | 0 | 0 | 0 | 0 | 0 | 0 | 0 | 0 | 0 | 0 | 0 | 0 | 0 |
| CaHDZ06 | 0.284 | 0.931 | 2.887 | 1.415 | 1.485 | 0.519 | 0.344 | 0.155 | 0.151 | 0 | 1.739 | 0.277 | 0.252 | 0.172 | 0.13 | 0 | 0 |
| CaHDZ07 | 0.45 | 1.056 | 0 | 0 | 0 | 0 | 0.706 | 0.965 | 1.649 | 0.261 | 0 | 0 | 0.2 | 0 | 0 | 0 | 0.354 |
| CaHDZ08 | 0 | 0.536 | 0 | 0 | 1.028 | 0.456 | 0.837 | 0 | 0.4 | 0 | 0 | 0 | 0 | 0 | 0 | 0 | 0 |
| CaHDZ09 | 0.541 | 6.942 | 1.678 | 0.834 | 1.739 | 1.402 | 0.596 | 1.014 | 0.731 | 3.141 | 1.212 | 3.491 | 2.976 | 1.84 | 3.87 | 2.256 | 3.298 |
| CaHDZ10 | 6.562 | 3.872 | 3.694 | 0.34 | 1.483 | 0.467 | 1.057 | 0.955 | 1.628 | 0.769 | 1.222 | 1.839 | 2.66 | 2.185 | 1.431 | 1.105 | 1.579 |
| CaHDZ11 | 7.796 | 0.745 | 0.382 | 0.337 | 0.551 | 0.623 | 0.145 | 0.138 | 0 | 0.118 | 1.515 | 6.68 | 4.003 | 2.274 | 2.466 | 1.051 | 1.354 |
| CaHDZ12 | 0.593 | 3.833 | 1.226 | 1.269 | 1.18 | 1.397 | 0.605 | 0.683 | 0.548 | 0.878 | 0.765 | 0.605 | 0.687 | 0.903 | 0.469 | 0.472 | 0.937 |
| CaHDZ13 | 196.238 | 176.871 | 214.672 | 75.488 | 54.795 | 54.328 | 43.791 | 46.273 | 55.189 | 50.351 | 209.442 | 129.991 | 139.455 | 172.364 | 126.534 | 110.346 | 98.947 |
| CaHDZ14 | 78.847 | 74.673 | 183.546 | 120.275 | 63.908 | 29.215 | 5.661 | 1.275 | 0.81 | 0.136 | 289.528 | 92.488 | 14.782 | 1.931 | 2.636 | 0.177 | 0.442 |
| CaHDZ16 | 0 | 0.293 | 0.292 | 0.535 | 2.715 | 1.233 | 0.917 | 1.524 | 0.21 | 1.074 | 0.369 | 0 | 0 | 0 | 0.369 | 0.287 | 0.33 |
| CaHDZ17 | 16.107 | 31.053 | 31.326 | 38.328 | 28.589 | 37.865 | 38.896 | 34.107 | 28.625 | 63.941 | 22.848 | 29.877 | 57.844 | 62.422 | 54.623 | 73.103 | 91.153 |
| CaHDZ18 | 10.718 | 14.12 | 21.754 | 14.943 | 14.239 | 14.311 | 10.752 | 10.855 | 12.325 | 13.826 | 19.187 | 14.228 | 16.135 | 15.774 | 16.257 | 15.331 | 11.682 |
| CaHDZ19 | 0 | 0.777 | 1.237 | 1.211 | 1.314 | 1.27 | 1.524 | 3.341 | 1.506 | 2.441 | 1.281 | 2.68 | 1.16 | 1.941 | 1.27 | 0.872 | 2.011 |
| CaHDZ20 | 31.856 | 77.389 | 48.109 | 37.936 | 50.155 | 37.47 | 28.344 | 15.856 | 9.698 | 11.124 | 69.606 | 48.637 | 81.991 | 44.508 | 44.807 | 24.369 | 24.056 |
| CaHDZ23 | 5.117 | 7.362 | 12.883 | 8.315 | 2.975 | 4.28 | 5.195 | 4.89 | 5.053 | 3.218 | 12.469 | 5.933 | 8.357 | 12.001 | 5.677 | 8.263 | 9.932 |
| CaHDZ24 | 1.49 | 3.993 | 6.46 | 19.282 | 19.558 | 26.689 | 17.771 | 8.595 | 7.027 | 9.741 | 2.371 | 2.61 | 10.076 | 8.351 | 15.566 | 25.867 | 35.506 |
| CaHDZ25 | 7.45 | 0.609 | 2.718 | 1.311 | 0.776 | 0.3 | 1.476 | 0.876 | 0.371 | 0.097 | 2.623 | 9.895 | 6.789 | 10.089 | 3.374 | 1.458 | 0.602 |
| CaHDZ26 | 0 | 0 | 0 | 0 | 0 | 0 | 0 | 0 | 0 | 0 | 0 | 0 | 0 | 0 | 0 | 0 | 0 |
| CaHDZ27 | 20.316 | 44.022 | 45.23 | 18.297 | 13.613 | 16.354 | 22.598 | 21.671 | 29.312 | 22.939 | 40.707 | 100.028 | 65.93 | 78.796 | 80.992 | 75.729 | 49.327 |
| CaHDZ28 | 13.095 | 2.565 | 15.203 | 23.214 | 13.187 | 13.315 | 15.914 | 10.185 | 15.729 | 15.719 | 16.657 | 6.499 | 5.02 | 5.675 | 9.682 | 10.215 | 13.989 |
| CaHDZ29 | 235.836 | 3 | 2.956 | 0 | 1.937 | 5.911 | 11.919 | 22.149 | 9.938 | 19.557 | 0.569 | 96.558 | 120.495 | 157.495 | 106.361 | 138.079 | 122.576 |
| CaHDZ30 | 40.356 | 546.054 | 756.626 | 2279.919 | 641.113 | 161.882 | 3.371 | 0.863 | 0.828 | 0.478 | 810.488 | 39.429 | 7.85 | 1.624 | 1.196 | 0.638 | 0.6 |
| CaHDZ31 | 0.994 | 0.201 | 0 | 0.264 | 2.728 | 0.571 | 0.482 | 0.934 | 0.765 | 3.757 | 0.122 | 0 | 0 | 0 | 0 | 0.105 | 0 |
| CaHDZ32 | 0 | 0 | 0 | 0 | 0 | 0 | 0 | 0 | 0 | 0 | 0 | 0.149 | 0 | 0 | 0 | 0 | 0 |
| CaHDZ34 | 0.334 | 0.984 | 0.986 | 0.625 | 0.701 | 0.707 | 0.44 | 1.066 | 0.571 | 0.756 | 1.371 | 2.911 | 3.769 | 1.322 | 2.003 | 0.66 | 0.872 |
| CaHDZ35 | 0 | 0 | 0 | 0 | 0 | 0 | 0 | 0 | 0 | 0 | 0 | 0 | 0 | 0 | 0 | 0 | 0 |
| CaHDZ37 | 17.115 | 55.853 | 47.332 | 35.663 | 35.481 | 38.236 | 46.527 | 40.033 | 39.223 | 50.761 | 48.844 | 69.806 | 54.044 | 43.834 | 67.213 | 64.6 | 62.61 |
| CaHDZ38 | 2.787 | 3.951 | 2.059 | 2.982 | 2.334 | 4.063 | 3.18 | 3.07 | 3.752 | 4.76 | 4.677 | 4.621 | 4.434 | 4.908 | 4.677 | 5.854 | 5.563 |
| CaHDZ39 | 0 | 0 | 0 | 0 | 0 | 0 | 0.448 | 0 | 0 | 0.342 | 0 | 0.357 | 0 | 0 | 0 | 0 | 0 |

**S4B: the transcriptome data (including raw data for expression(B) under salt stress)**

| gene-id | VPC-4h | VPY-4h | VPC-58h | VPY-58h |
| --- | --- | --- | --- | --- |
| CaHDZ01 | 20.07427 | 30.44525 | 23.58897 | 30.24761 |
| CaHDZ02 | 0.023849 | 0.06935 | 0.011095 | 0.044185 |
| CaHDZ03 | 8.376611 | 36.05461 | 0.404024 | 3.557797 |
| CaHDZ04 | 6.589795 | 11.57689 | 3.695221 | 3.993189 |
| CaHDZ05 | 2.505632 | 3.87391 | 8.704286 | 7.498849 |
| CaHDZ06 | 2.492019 | 1.943772 | 8.516125 | 5.954271 |
| CaHDZ07 | 15.22755 | 13.95549 | 34.02595 | 42.63672 |
| CaHDZ08 | 8.996168 | 11.59125 | 15.3431 | 14.61232 |
| CaHDZ09 | 0.150856 | 1.589425 | 0.984502 | 1.585086 |
| CaHDZ10 | 12.73264 | 12.94071 | 19.21466 | 22.89664 |
| CaHDZ11 | 6.355439 | 5.628783 | 4.989364 | 6.157741 |
| CaHDZ12 | 1.725643 | 2.432257 | 2.479579 | 2.000752 |
| CaHDZ13 | 24.17414 | 58.16395 | 21.33535 | 30.10167 |
| CaHDZ15 | 4.90463 | 5.018599 | 5.571209 | 5.475722 |
| CaHDZ16 | 4.774365 | 6.124279 | 11.79313 | 12.15832 |
| CaHDZ17 | 19.34486 | 18.68101 | 7.793341 | 13.79402 |
| CaHDZ18 | 0.477089 | 0.399895 | 0.338832 | 0.620474 |
| CaHDZ19 | 54.16464 | 77.6131 | 23.05414 | 33.41003 |
| CaHDZ20 | 0 | 0.063718 | 0.029943 | 0 |
| CaHDZ21 | 108.8369 | 86.87736 | 56.39572 | 45.12265 |
| CaHDZ25 | 78.68192 | 109.1379 | 39.01917 | 49.76128 |
| CaHDZ26 | 0.105319 | 0.184458 | 0.04174 | 0.832949 |
| CaHDZ27 | 1.041734 | 0.601976 | 3.571135 | 0.619295 |
| CaHDZ28 | 0.385327 | 0.887146 | 0.839824 | 1.071836 |
| CaHDZ29 | 0.012661 | 0 | 0 | 0 |
| CaHDZ30 | 0.69793 | 1.641276 | 0.008105 | 0.305733 |
| CaHDZ31 | 0.270153 | 0.149848 | 1.044071 | 1.16049 |
| CaHDZ32 | 6.7759 | 8.462889 | 14.00236 | 9.884363 |
| CaHDZ33 | 1.683169 | 1.756919 | 1.389685 | 0.771778 |
| CaHDZ34 | 3.456528 | 4.658026 | 14.0924 | 15.16502 |
| CaHDZ35 | 32.21394 | 60.563 | 11.29995 | 13.09908 |
| CaHDZ36 | 16.84552 | 19.88946 | 13.89454 | 15.12923 |
| CaHDZ37 | 0 | 0 | 0.003551 | 0 |
| CaHDZ38 | 37.93931 | 31.57265 | 111.1361 | 68.58135 |
| CaHDZ39 | 5.82059 | 6.51801 | 10.18184 | 10.45101 |
| CaHDZ40 | 0 | 0.012531 | 0 | 0.022919 |
