# Supplementary material for: Genome-Wide Characterization and Expression Analysis of the HD-ZIP Gene Family in Response to Salt Stress in Pepper
Source: Int J Genomics. 2021 Jan 25;2021:8105124. doi: 10.1155/2021/8105124 (PMC7869415; doi:10.1155/2021/8105124)
Supplement: Supplementary 5 — Table S5: the specific primer for qRT-PCRof each CaHD-ZIP gene. [file 8105124.f5.docx]

| **Table5.Primers used for qRT-PCR** | | | | | |
| --- | --- | --- | --- | --- | --- |
| *CaHDZ21F* | CAGGGGAAAGTGGTTGGAGG |  |  |  |  |
| *CaHDZ21R* | TAGCCGGTCCTCAGTCTTCA |  |  |  |  |
| *CaHDZ39F* | TAGAGCTTGTGGCCTGGTTG |  |  |  |  |
| *CaHDZ39R* | AACAGCCAGAAGTCACGAGG |  |  |  |  |
| *CaHDZ33*F | *GCGCTTTCATCTCCGAACAG* |  |  |  |  |
| *CaHDZ33*R | *CGCGTCATCTTCATCACTTGC* |  |  |  |  |
| *CaHDZ32F* | AGTACCTACGCCGCTTGTTC |  |  |  |  |
| *CaHDZ32R* | TGGCCTTCTTCTACAGCGTG |  |  |  |  |
| *CaHDZ25F* | CGGCTTTGGATCCGAGAGAA |  |  |  |  |
| *CaHDZ25R* | TCCATCGAGCACGACGATTT |  |  |  |  |
| *CaHDZ35F* | AGAGGCCATTTTTCCCAGCA |  |  |  |  |
| *CaHDZ35R* | TGTCTAGGCTGCAATCCAAGT |  |  |  |  |
| *CaHDZ4F* | CCTCACCATGTGCCCTTCAT |  |  |  |  |
| *CaHDZ4R* | AGGATGTAGTGCATTGGCCC |  |  |  |  |
| *CaHDZ3F* | CTGCAGCCAAGACAAGTAGC |  |  |  |  |
| *CaHDZ3R* | CAGTGCCACCCTTTTCAGTT |  |  |  |  |
| *CaHDZ10F* | CTGCGCCGAACTCGTATTTG |  |  |  |  |
| *CaHDZ10R* | GACTGGACGCTTCCTTTCCA |  |  |  |  |
