# Supplementary material for: Genome-Wide Characterization and Expression Analysis of the HD-ZIP Gene Family in Response to Salt Stress in Pepper
Source: Int J Genomics. 2021 Jan 25;2021:8105124. doi: 10.1155/2021/8105124 (PMC7869415; doi:10.1155/2021/8105124)
Supplement: Supplementary 6 — Table S6: pepper HD-ZIP gene promoter function element. [file 8105124.f6.docx]

| Pepper HD-ZIP gene promoter function element. | | | | | | | |  |
| --- | --- | --- | --- | --- | --- | --- | --- | --- |
| CaHDZ01 | CGTCA-motif | CGTCA | 1361 | 5 | - | Hordeum vulgare | MeJA |  |
| CaHDZ01 | CGTCA-motif | CGTCA | 1771 | 5 | + | Hordeum vulgare | MeJA |  |
| CaHDZ01 | P-box | CCTTTTG | 728 | 7 | + | Oryza sativa | Gibberellin | |
| CaHDZ01 | AE-box | AGAAACTT | 525 | 8 | - | Arabidopsis thaliana | Light |  |
| CaHDZ01 | Box 4 | ATTAAT | 153 | 6 | + | Petroselinum crispum | Light |  |
| CaHDZ01 | Box 4 | ATTAAT | 362 | 6 | + | Petroselinum crispum | Light |  |
| CaHDZ01 | Box 4 | ATTAAT | 685 | 6 | + | Petroselinum crispum | Light |  |
| CaHDZ01 | Box 4 | ATTAAT | 821 | 6 | + | Petroselinum crispum | Light |  |
| CaHDZ01 | TGACG-motif | TGACG | 1361 | 5 | + | Hordeum vulgare | MeJA |  |
| CaHDZ01 | TGACG-motif | TGACG | 1771 | 5 | - | Hordeum vulgare | MeJA |  |
| CaHDZ01 | GT1-motif | GGTTAA | 1028 | 6 | - | Arabidopsis thaliana | Light |  |
| CaHDZ01 | GT1-motif | GGTTAA | 1620 | 6 | + | Arabidopsis thaliana | Light |  |
| CaHDZ01 | ARE | AAACCA | 1371 | 6 | - | Zea mays | Anaerobic induction | |
| CaHDZ01 | ARE | AAACCA | 1843 | 6 | - | Zea mays | Anaerobic induction | |
| CaHDZ01 | TCA-element | CCATCTTTTT | 919 | 9 | + | Nicotiana tabacum | Salicylic acid | |
| CaHDZ01 | TCA-element | CCATCTTTTT | 1952 | 9 | + | Nicotiana tabacum | Salicylic acid | |
| CaHDZ01 | TCT-motif | TCTTAC | 773 | 6 | - | Arabidopsis thaliana | Light |  |
| CaHDZ01 | TCT-motif | TCTTAC | 1474 | 6 | + | Arabidopsis thaliana | Light |  |
| CaHDZ01 | TCT-motif | TCTTAC | 1630 | 6 | - | Arabidopsis thaliana | Light |  |
| CaHDZ03 | I-box | cCATATCCAAT | 1018 | 10 | - | Flaveria trinervia | Light |  |
| CaHDZ03 | Box 4 | ATTAAT | 728 | 6 | + | Petroselinum crispum | Light |  |
| CaHDZ03 | Box 4 | ATTAAT | 735 | 6 | + | Petroselinum crispum | Light |  |
| CaHDZ03 | Box 4 | ATTAAT | 1835 | 6 | - | Petroselinum crispum | Light |  |
| CaHDZ03 | CCAAT-box | CAACGG | 192 | 6 | + | Hordeum vulgare | MYB binding site | |
| CaHDZ03 | TC-rich repeats | ATTCTCTAAC | 409 | 9 | + | Nicotiana tabacum | Defense and stress | |
| CaHDZ03 | TC-rich repeats | GTTTTCTTAC | 439 | 9 | - | Nicotiana tabacum | Defense and stress | |
| CaHDZ03 | G-Box | CACGTG | 1355 | 6 | - | Pisum sativum | Light |  |
| CaHDZ03 | G-box | CCACGTAA | 968 | 8 | + | Brassica napus | Light |  |
| CaHDZ03 | G-box | TACGTG | 969 | 6 | - | Arabidopsis thaliana | Light |  |
| CaHDZ03 | G-box | TACGTG | 983 | 6 | + | Arabidopsis thaliana | Light |  |
| CaHDZ03 | G-box | CACGTG | 1355 | 6 | - | Arabidopsis thaliana | Light |  |
| CaHDZ03 | GT1-motif | GGTTAA | 1507 | 6 | + | Arabidopsis thaliana | Light |  |
| CaHDZ03 | ARE | AAACCA | 358 | 6 | + | Zea mays | Anaerobic induction | |
| CaHDZ03 | ARE | AAACCA | 1975 | 6 | + | Zea mays | Anaerobic induction | |
| CaHDZ03 | MBS | CAACTG | 685 | 6 | + | Arabidopsis thaliana | MYB binding site | |
| CaHDZ03 | TGA-element | AACGAC | 584 | 6 | + | Brassica oleracea | Auxin |  |
| CaHDZ03 | TCT-motif | TCTTAC | 439 | 6 | - | Arabidopsis thaliana | Light |  |
| CaHDZ03 | TCT-motif | TCTTAC | 1111 | 6 | + | Arabidopsis thaliana | Light |  |
| CaHDZ03 | TCT-motif | TCTTAC | 1819 | 6 | - | Arabidopsis thaliana | Light |  |
| CaHDZ03 | ABRE | ACGTG | 969 | 5 | - | Arabidopsis thaliana | Abscisic acid | |
| CaHDZ03 | ABRE | ACGTG | 984 | 5 | + | Arabidopsis thaliana | Abscisic acid | |
| CaHDZ03 | ABRE | GACACGTGGC | 1353 | 9 | + | Triticum aestivum | Abscisic acid | |
| CaHDZ03 | ABRE | CACGTG | 1355 | 6 | - | Arabidopsis thaliana | Abscisic acid | |
| CaHDZ03 | ABRE | ACGTG | 1356 | 5 | + | Arabidopsis thaliana | Abscisic acid | |
| CaHDZ04 | CGTCA-motif | CGTCA | 716 | 5 | - | Hordeum vulgare | MeJA |  |
| CaHDZ04 | Box 4 | ATTAAT | 766 | 6 | + | Petroselinum crispum | Light |  |
| CaHDZ04 | Box 4 | ATTAAT | 970 | 6 | + | Petroselinum crispum | Light |  |
| CaHDZ04 | Box 4 | ATTAAT | 974 | 6 | + | Petroselinum crispum | Light |  |
| CaHDZ04 | Box 4 | ATTAAT | 1013 | 6 | - | Petroselinum crispum | Light |  |
| CaHDZ04 | TGACG-motif | TGACG | 716 | 5 | + | Hordeum vulgare | MeJA |  |
| CaHDZ04 | chs-CMA1a | TTACTTAA | 1495 | 8 | + | Daucus carota | Light |  |
| CaHDZ04 | AT1-motif | AATTATTTTTTATT | 1141 | 13 | + | Solanum tuberosum | Light |  |
| CaHDZ04 | G-box | TACGTG | 370 | 6 | - | Arabidopsis thaliana | Light |  |
| CaHDZ04 | GT1-motif | GGTTAA | 1530 | 6 | + | Arabidopsis thaliana | Light |  |
| CaHDZ04 | GT1-motif | GGTTAA | 1532 | 6 | - | Arabidopsis thaliana | Light |  |
| CaHDZ04 | ARE | AAACCA | 569 | 6 | - | Zea mays | Anaerobic induction | |
| CaHDZ04 | TCA-element | CCATCTTTTT | 169 | 9 | + | Nicotiana tabacum | Salicylic acid | |
| CaHDZ04 | TCA-element | TCAGAAGAGG | 1719 | 9 | - | Brassica oleracea | Salicylic acid | |
| CaHDZ04 | TCT-motif | TCTTAC | 321 | 6 | + | Arabidopsis thaliana | Light |  |
| CaHDZ04 | TCT-motif | TCTTAC | 1433 | 6 | - | Arabidopsis thaliana | Light |  |
| CaHDZ04 | TCT-motif | TCTTAC | 1493 | 6 | + | Arabidopsis thaliana | Light |  |
| CaHDZ04 | ABRE | ACGTG | 370 | 5 | - | Arabidopsis thaliana | Abscisic acid | |
| CaHDZ05 | ACE | CTAACGTATT | 1585 | 9 | - | Petroselinum crispum | Light |  |
| CaHDZ05 | WUN-motif | AAATTTCCT | 1511 | 9 | - | Brassica oleracea | Wound |  |
| CaHDZ05 | 3-AF1 binding site | TAAGAGAGGAA | 1356 | 10 | + | Solanum tuberosum | Light |  |
| CaHDZ05 | TC-rich repeats | GTTTTCTTAC | 1480 | 9 | + | Nicotiana tabacum | Defense and stress | |
| CaHDZ05 | P-box | CCTTTTG | 125 | 7 | - | Oryza sativa | Gibberellin | |
| CaHDZ05 | I-box | AGATAAGG | 1104 | 8 | + | Triticum aestivum | Light |  |
| CaHDZ05 | Box 4 | ATTAAT | 484 | 6 | + | Petroselinum crispum | Light |  |
| CaHDZ05 | Box 4 | ATTAAT | 1500 | 6 | - | Petroselinum crispum | Light |  |
| CaHDZ05 | G-Box | CACGTG | 1213 | 6 | - | Pisum sativum | Light |  |
| CaHDZ05 | GATA-motif | AAGGATAAGG | 1102 | 9 | + | Solanum tuberosum | Light |  |
| CaHDZ05 | G-box | tgACACGTGGCA | 1210 | 11 | - | Lycopersicon esculentum | Light |  |
| CaHDZ05 | G-box | ACACGTGGC | 1211 | 9 | - | Brassica napus | Light |  |
| CaHDZ05 | G-box | CACGTG | 1213 | 6 | - | Arabidopsis thaliana | Light |  |
| CaHDZ05 | GT1-motif | GGTTAA | 1302 | 6 | - | Arabidopsis thaliana | Light |  |
| CaHDZ05 | MRE | AACCTAA | 1937 | 7 | + | Petroselinum crispum | MYB binding site | |
| CaHDZ05 | ARE | AAACCA | 442 | 6 | + | Zea mays | Anaerobic induction | |
| CaHDZ05 | ARE | AAACCA | 1592 | 6 | - | Zea mays | Anaerobic induction | |
| CaHDZ05 | GA-motif | ATAGATAA | 1036 | 8 | - | Arabidopsis thaliana | Light |  |
| CaHDZ05 | ABRE | GCAACGTGTC | 1211 | 9 | + | Hordeum vulgare | Abscisic acid | |
| CaHDZ05 | ABRE | CACGTG | 1213 | 6 | - | Arabidopsis thaliana | Abscisic acid | |
| CaHDZ05 | ABRE | ACGTG | 1214 | 5 | + | Arabidopsis thaliana | Abscisic acid | |
| CaHDZ06 | CGTCA-motif | CGTCA | 606 | 5 | - | Hordeum vulgare | MeJA |  |
| CaHDZ06 | P-box | CCTTTTG | 1766 | 7 | - | Oryza sativa | Gibberellin | |
| CaHDZ06 | AE-box | AGAAACAA | 1574 | 8 | + | Arabidopsis thaliana | Light |  |
| CaHDZ06 | I-box | gGATAAGGTG | 1523 | 9 | - | Zea mays | Light |  |
| CaHDZ06 | Box 4 | ATTAAT | 991 | 6 | + | Petroselinum crispum | Light |  |
| CaHDZ06 | Box 4 | ATTAAT | 1189 | 6 | - | Petroselinum crispum | Light |  |
| CaHDZ06 | Box 4 | ATTAAT | 1462 | 6 | - | Petroselinum crispum | Light |  |
| CaHDZ06 | Box 4 | ATTAAT | 1713 | 6 | - | Petroselinum crispum | Light |  |
| CaHDZ06 | TGACG-motif | TGACG | 606 | 5 | + | Hordeum vulgare | MeJA |  |
| CaHDZ06 | WUN-motif | AAATTTCCT | 552 | 9 | - | Brassica oleracea | Wound |  |
| CaHDZ06 | AT1-motif | AATTATTTTTTATT | 935 | 13 | - | Solanum tuberosum | Light |  |
| CaHDZ06 | GATA-motif | AAGATAAGATT | 1046 | 10 | + | Arabidopsis thaliana | Light |  |
| CaHDZ06 | G-box | CACGAC | 1081 | 6 | - | Zea mays | Light |  |
| CaHDZ06 | GT1-motif | GGTTAA | 443 | 6 | + | Arabidopsis thaliana | Light |  |
| CaHDZ06 | ARE | AAACCA | 1580 | 6 | + | Zea mays | Anaerobic induction | |
| CaHDZ06 | ARE | AAACCA | 1595 | 6 | - | Zea mays | Anaerobic induction | |
| CaHDZ06 | GA-motif | ATAGATAA | 128 | 8 | + | Arabidopsis thaliana | Light |  |
| CaHDZ06 | GC-motif | CCCCCG | 782 | 6 | + | Zea mays | Anoxic specific inducibility | |
| CaHDZ06 | TCA-element | CCATCTTTTT | 1148 | 9 | - | Nicotiana tabacum | Salicylic acid | |
| CaHDZ06 | TGA-element | AACGAC | 275 | 6 | - | Brassica oleracea | Auxin |  |
| CaHDZ06 | TGA-element | AACGAC | 1542 | 6 | + | Brassica oleracea | Auxin |  |
| CaHDZ07 | AT1-motif | AATTATTTTTTATT | 363 | 13 | - | Solanum tuberosum | Light |  |
| CaHDZ07 | AT1-motif | AATTATTTTTTATT | 479 | 13 | + | Solanum tuberosum | Light |  |
| CaHDZ07 | AT1-motif | AATTATTTTTTATT | 797 | 13 | - | Solanum tuberosum | Light |  |
| CaHDZ07 | 3-AF1 binding site | TAAGAGAGGAA | 1612 | 10 | + | Solanum tuberosum | Light |  |
| CaHDZ07 | MBSI | aaaAaaC(G/C)GTTA | 1555 | 10.5 | - | Petunia hybrida | MYB binding site | |
| CaHDZ07 | Box 4 | ATTAAT | 285 | 6 | + | Petroselinum crispum | Light |  |
| CaHDZ07 | Box 4 | ATTAAT | 304 | 6 | + | Petroselinum crispum | Light |  |
| CaHDZ07 | Box 4 | ATTAAT | 360 | 6 | + | Petroselinum crispum | Light |  |
| CaHDZ07 | Box 4 | ATTAAT | 442 | 6 | + | Petroselinum crispum | Light |  |
| CaHDZ07 | Box 4 | ATTAAT | 900 | 6 | + | Petroselinum crispum | Light |  |
| CaHDZ07 | Box 4 | ATTAAT | 1035 | 6 | - | Petroselinum crispum | Light |  |
| CaHDZ07 | TCCC-motif | TCTCCCT | 1460 | 7 | - | Spinacia oleracea | Light |  |
| CaHDZ07 | GT1-motif | GGTTAA | 969 | 6 | + | Arabidopsis thaliana | Light |  |
| CaHDZ07 | ARE | AAACCA | 1355 | 6 | + | Zea mays | Anaerobic induction | |
| CaHDZ07 | ARE | AAACCA | 1402 | 6 | - | Zea mays | Anaerobic induction | |
| CaHDZ07 | ARE | AAACCA | 1800 | 6 | - | Zea mays | Anaerobic induction | |
| CaHDZ07 | LTR | CCGAAA | 845 | 6 | - | Hordeum vulgare | Low temperature | |
| CaHDZ08 | CGTCA-motif | CGTCA | 285 | 5 | + | Hordeum vulgare | MeJA |  |
| CaHDZ08 | 3-AF1 binding site | TAAGAGAGGAA | 1986 | 10 | + | Solanum tuberosum | Light |  |
| CaHDZ08 | AE-box | AGAAACAA | 1464 | 8 | + | Arabidopsis thaliana | Light |  |
| CaHDZ08 | Box 4 | ATTAAT | 372 | 6 | + | Petroselinum crispum | Light |  |
| CaHDZ08 | TCCC-motif | TCTCCCT | 1555 | 7 | + | Spinacia oleracea | Light |  |
| CaHDZ08 | TGACG-motif | TGACG | 285 | 5 | - | Hordeum vulgare | MeJA |  |
| CaHDZ08 | TATC-box | TATCCCA | 1675 | 7 | + | Oryza sativa | Gibberellin | |
| CaHDZ08 | chs-CMA1a | TTACTTAA | 418 | 8 | - | Daucus carota | Light |  |
| CaHDZ08 | TC-rich repeats | GTTTTCTTAC | 36 | 9 | + | Nicotiana tabacum | Defense and stress | |
| CaHDZ08 | TC-rich repeats | GTTTTCTTAC | 1118 | 9 | + | Nicotiana tabacum | Defense and stress | |
| CaHDZ08 | GATA-motif | GATAGGG | 1489 | 7 | - | Pisum sativum | Light |  |
| CaHDZ08 | G-box | GCCACGTGGA | 240 | 9 | + | Arabidopsis thaliana | Light |  |
| CaHDZ08 | G-box | TAACACGTAG | 860 | 9 | + | Brassica oleracea | Light |  |
| CaHDZ08 | G-box | TACGTG | 863 | 6 | - | Arabidopsis thaliana | Light |  |
| CaHDZ08 | G-box | TAACACGTAG | 936 | 9 | - | Brassica oleracea | Light |  |
| CaHDZ08 | GT1-motif | GGTTAA | 907 | 6 | - | Arabidopsis thaliana | Light |  |
| CaHDZ08 | GT1-motif | GGTTAA | 915 | 6 | + | Arabidopsis thaliana | Light |  |
| CaHDZ08 | TCA-element | CCATCTTTTT | 1923 | 9 | + | Nicotiana tabacum | Salicylic acid | |
| CaHDZ08 | TCT-motif | TCTTAC | 40 | 6 | + | Arabidopsis thaliana | Light |  |
| CaHDZ08 | TCT-motif | TCTTAC | 407 | 6 | + | Arabidopsis thaliana | Light |  |
| CaHDZ08 | ABRE | ACGTG | 863 | 5 | - | Arabidopsis thaliana | Abscisic acid | |
| CaHDZ09 | CGTCA-motif | CGTCA | 1982 | 5 | + | Hordeum vulgare | MeJA |  |
| CaHDZ09 | Gap-box | CAAATGAA(A/G)A | 366 | 9 | - | Arabidopsis thaliana | Light |  |
| CaHDZ09 | GARE-motif | TCTGTTG | 529 | 7 | + | Brassica oleracea | Gibberellin | |
| CaHDZ09 | GARE-motif | TCTGTTG | 849 | 7 | - | Brassica oleracea | Gibberellin | |
| CaHDZ09 | Box 4 | ATTAAT | 807 | 6 | + | Petroselinum crispum | Light |  |
| CaHDZ09 | Box 4 | ATTAAT | 939 | 6 | + | Petroselinum crispum | Light |  |
| CaHDZ09 | Box 4 | ATTAAT | 1263 | 6 | - | Petroselinum crispum | Light |  |
| CaHDZ09 | TGACG-motif | TGACG | 1982 | 5 | - | Hordeum vulgare | MeJA |  |
| CaHDZ09 | G-Box | CACGTG | 1141 | 6 | - | Pisum sativum | Light |  |
| CaHDZ09 | G-box | TACGTG | 672 | 6 | - | Arabidopsis thaliana | Light |  |
| CaHDZ09 | G-box | CACGAC | 1043 | 6 | - | Zea mays | Light |  |
| CaHDZ09 | G-box | GCCACGTGGA | 1139 | 9 | + | Arabidopsis thaliana | Light |  |
| CaHDZ09 | G-box | CACGTG | 1141 | 6 | - | Arabidopsis thaliana | Light |  |
| CaHDZ09 | GT1-motif | GGTTAA | 456 | 6 | - | Arabidopsis thaliana | Light |  |
| CaHDZ09 | GT1-motif | GGTTAA | 1391 | 6 | + | Arabidopsis thaliana | Light |  |
| CaHDZ09 | ARE | AAACCA | 968 | 6 | + | Zea mays | Anaerobic induction | |
| CaHDZ09 | ARE | AAACCA | 1702 | 6 | - | Zea mays | Anaerobic induction | |
| CaHDZ09 | GA-motif | ATAGATAA | 418 | 8 | + | Arabidopsis thaliana | Light |  |
| CaHDZ09 | chs-CMA2a | TCACTTGA | 766 | 8 | + | Petroselinum crispum | Light |  |
| CaHDZ09 | chs-CMA2a | TCACTTGA | 800 | 8 | + | Petroselinum crispum | Light |  |
| CaHDZ09 | TCT-motif | TCTTAC | 914 | 6 | + | Arabidopsis thaliana | Light |  |
| CaHDZ09 | TCT-motif | TCTTAC | 1049 | 6 | - | Arabidopsis thaliana | Light |  |
| CaHDZ09 | TGA-element | AACGAC | 788 | 6 | - | Brassica oleracea | Auxin |  |
| CaHDZ09 | TGA-element | AACGAC | 1462 | 6 | - | Brassica oleracea | Auxin |  |
| CaHDZ09 | ABRE | ACGTG | 672 | 5 | - | Arabidopsis thaliana | Abscisic acid | |
| CaHDZ09 | ABRE | CACGTG | 1141 | 6 | - | Arabidopsis thaliana | Abscisic acid | |
| CaHDZ09 | ABRE | ACGTG | 1142 | 5 | + | Arabidopsis thaliana | Abscisic acid | |
| CaHDZ09 | ATCT-motif | AATCTAATCC | 72 | 9 | - | Pisum sativum | Light |  |
| CaHDZ10 | TC-rich repeats | ATTCTCTAAC | 1434 | 9 | + | Nicotiana tabacum | Defense and stress | |
| CaHDZ10 | I-box | atGATAAGGTC | 939 | 10 | - | Helianthus annuus | Light |  |
| CaHDZ10 | Box 4 | ATTAAT | 44 | 6 | + | Petroselinum crispum | Light |  |
| CaHDZ10 | Box 4 | ATTAAT | 1204 | 6 | - | Petroselinum crispum | Light |  |
| CaHDZ10 | Box 4 | ATTAAT | 1470 | 6 | - | Petroselinum crispum | Light |  |
| CaHDZ10 | TCCC-motif | TCTCCCT | 458 | 7 | - | Spinacia oleracea | Light |  |
| CaHDZ10 | G-Box | CACGTT | 1171 | 6 | + | Pisum sativum | Light |  |
| CaHDZ10 | GT1-motif | GGTTAAT | 619 | 7 | + | Avena sativa | Light |  |
| CaHDZ10 | GT1-motif | GGTTAAT | 731 | 7 | - | Avena sativa | Light |  |
| CaHDZ10 | GT1-motif | GGTTAA | 732 | 6 | - | Arabidopsis thaliana | Light |  |
| CaHDZ10 | GT1-motif | GGTTAA | 982 | 6 | - | Arabidopsis thaliana | Light |  |
| CaHDZ10 | GT1-motif | GGTTAA | 1647 | 6 | + | Arabidopsis thaliana | Light |  |
| CaHDZ10 | ARE | AAACCA | 907 | 6 | - | Zea mays | Anaerobic induction | |
| CaHDZ10 | ARE | AAACCA | 1933 | 6 | - | Zea mays | Anaerobic induction | |
| CaHDZ10 | MBS | CAACTG | 5 | 6 | + | Arabidopsis thaliana | MYB binding site | |
| CaHDZ10 | MBS | CAACTG | 20 | 6 | - | Arabidopsis thaliana | MYB binding site | |
| CaHDZ10 | MBS | CAACTG | 703 | 6 | + | Arabidopsis thaliana | MYB binding site | |
| CaHDZ10 | TCA-element | TCAGAAGAGG | 823 | 9 | + | Brassica oleracea | Salicylic acid | |
| CaHDZ10 | TCT-motif | TCTTAC | 510 | 6 | + | Arabidopsis thaliana | Light |  |
| CaHDZ10 | ABRE | TACGGTC | 939 | 7 | - | Arabidopsis thaliana | Abscisic acid | |
| CaHDZ10 | ABRE | ACGTG | 1171 | 5 | - | Arabidopsis thaliana | Abscisic acid | |
| CaHDZ11 | chs-CMA1a | TTACTTAA | 1722 | 8 | + | Daucus carota | Light |  |
| CaHDZ11 | P-box | CCTTTTG | 1788 | 7 | - | Oryza sativa | Gibberellin | |
| CaHDZ11 | AE-box | AGAAACAA | 1550 | 8 | - | Arabidopsis thaliana | Light |  |
| CaHDZ11 | Box 4 | ATTAAT | 883 | 6 | + | Petroselinum crispum | Light |  |
| CaHDZ11 | Box 4 | ATTAAT | 914 | 6 | + | Petroselinum crispum | Light |  |
| CaHDZ11 | Box 4 | ATTAAT | 1142 | 6 | - | Petroselinum crispum | Light |  |
| CaHDZ11 | Box 4 | ATTAAT | 1657 | 6 | - | Petroselinum crispum | Light |  |
| CaHDZ11 | TCCC-motif | TCTCCCT | 896 | 7 | - | Spinacia oleracea | Light |  |
| CaHDZ11 | TCCC-motif | TCTCCCT | 1633 | 7 | + | Spinacia oleracea | Light |  |
| CaHDZ11 | GATA-motif | AAGATAAGATT | 683 | 10 | + | Arabidopsis thaliana | Light |  |
| CaHDZ11 | GT1-motif | GGTTAA | 395 | 6 | + | Arabidopsis thaliana | Light |  |
| CaHDZ11 | LAMP-element | CTTTATCA | 1397 | 8 | + | Pisum sativum | Light |  |
| CaHDZ11 | LAMP-element | CTTTATCA | 1944 | 8 | - | Pisum sativum | Light |  |
| CaHDZ11 | ARE | AAACCA | 1005 | 6 | + | Zea mays | Anaerobic induction | |
| CaHDZ11 | ARE | AAACCA | 1486 | 6 | + | Zea mays | Anaerobic induction | |
| CaHDZ11 | ARE | AAACCA | 1591 | 6 | - | Zea mays | Anaerobic induction | |
| CaHDZ11 | ARE | AAACCA | 1952 | 6 | - | Zea mays | Anaerobic induction | |
| CaHDZ11 | TCA-element | CCATCTTTTT | 177 | 9 | - | Nicotiana tabacum | Salicylic acid | |
| CaHDZ11 | TCT-motif | TCTTAC | 686 | 6 | - | Arabidopsis thaliana | Light |  |
| CaHDZ12 | CGTCA-motif | CGTCA | 28 | 5 | + | Hordeum vulgare | MeJA |  |
| CaHDZ12 | CGTCA-motif | CGTCA | 255 | 5 | - | Hordeum vulgare | MeJA |  |
| CaHDZ12 | CGTCA-motif | CGTCA | 1008 | 5 | - | Hordeum vulgare | MeJA |  |
| CaHDZ12 | CGTCA-motif | CGTCA | 1333 | 5 | + | Hordeum vulgare | MeJA |  |
| CaHDZ12 | TC-rich repeats | ATTCTCTAAC | 888 | 9 | - | Nicotiana tabacum | Defense and stress | |
| CaHDZ12 | TC-rich repeats | ATTCTCTAAC | 1708 | 9 | - | Nicotiana tabacum | Defense and stress | |
| CaHDZ12 | P-box | CCTTTTG | 708 | 7 | + | Oryza sativa | Gibberellin | |
| CaHDZ12 | AE-box | AGAAACAA | 835 | 8 | - | Arabidopsis thaliana | Light |  |
| CaHDZ12 | Box 4 | ATTAAT | 488 | 6 | + | Petroselinum crispum | Light |  |
| CaHDZ12 | Box 4 | ATTAAT | 750 | 6 | + | Petroselinum crispum | Light |  |
| CaHDZ12 | TGACG-motif | TGACG | 28 | 5 | - | Hordeum vulgare | MeJA |  |
| CaHDZ12 | TGACG-motif | TGACG | 255 | 5 | + | Hordeum vulgare | MeJA |  |
| CaHDZ12 | TGACG-motif | TGACG | 1008 | 5 | + | Hordeum vulgare | MeJA |  |
| CaHDZ12 | TGACG-motif | TGACG | 1333 | 5 | - | Hordeum vulgare | MeJA |  |
| CaHDZ12 | ARE | AAACCA | 1234 | 6 | + | Zea mays | Anaerobic induction | |
| CaHDZ12 | ARE | AAACCA | 1627 | 6 | + | Zea mays | Anaerobic induction | |
| CaHDZ12 | ARE | AAACCA | 1839 | 6 | - | Zea mays | Anaerobic induction | |
| CaHDZ12 | CCAAT-box | CAACGG | 955 | 6 | - | Hordeum vulgare | MYB binding site | |
| CaHDZ12 | MBS | CAACTG | 1087 | 6 | + | Arabidopsis thaliana | MYB binding site | |
| CaHDZ12 | TCA-element | CCATCTTTTT | 1526 | 9 | + | Nicotiana tabacum | Salicylic acid | |
| CaHDZ12 | TCA-element | CCATCTTTTT | 1849 | 9 | - | Nicotiana tabacum | Salicylic acid | |
| CaHDZ12 | TCT-motif | TCTTAC | 1101 | 6 | + | Arabidopsis thaliana | Light |  |
| CaHDZ12 | TGA-element | AACGAC | 1485 | 6 | - | Brassica oleracea | Auxin |  |
| CaHDZ13 | AT1-motif | AATTATTTTTTATT | 1342 | 13 | + | Solanum tuberosum | Light |  |
| CaHDZ13 | AE-box | AGAAACAA | 1659 | 8 | + | Arabidopsis thaliana | Light |  |
| CaHDZ13 | Box 4 | ATTAAT | 192 | 6 | + | Petroselinum crispum | Light |  |
| CaHDZ13 | Box 4 | ATTAAT | 1131 | 6 | - | Petroselinum crispum | Light |  |
| CaHDZ13 | Box 4 | ATTAAT | 1155 | 6 | - | Petroselinum crispum | Light |  |
| CaHDZ13 | Box 4 | ATTAAT | 1377 | 6 | - | Petroselinum crispum | Light |  |
| CaHDZ13 | Box 4 | ATTAAT | 1409 | 6 | - | Petroselinum crispum | Light |  |
| CaHDZ13 | Box 4 | ATTAAT | 1456 | 6 | - | Petroselinum crispum | Light |  |
| CaHDZ13 | Box 4 | ATTAAT | 1460 | 6 | - | Petroselinum crispum | Light |  |
| CaHDZ13 | Box 4 | ATTAAT | 1714 | 6 | - | Petroselinum crispum | Light |  |
| CaHDZ13 | TCCC-motif | TCTCCCT | 1531 | 7 | - | Spinacia oleracea | Light |  |
| CaHDZ13 | TCCC-motif | TCTCCCT | 1968 | 7 | + | Spinacia oleracea | Light |  |
| CaHDZ13 | GTGGC-motif | GATTCTGTGGC | 691 | 10 | - | Spinacia oleracea | Light |  |
| CaHDZ13 | G-box | CACGAC | 548 | 6 | - | Zea mays | Light |  |
| CaHDZ13 | G-box | CACGTC | 809 | 6 | + | Zea mays | Light |  |
| CaHDZ13 | GT1-motif | GGTTAAT | 293 | 7 | + | Avena sativa | Light |  |
| CaHDZ13 | GT1-motif | GGTTAA | 886 | 6 | + | Arabidopsis thaliana | Light |  |
| CaHDZ13 | ARE | AAACCA | 125 | 6 | + | Zea mays | Anaerobic induction | |
| CaHDZ13 | TCA-element | CCATCTTTTT | 1538 | 9 | - | Nicotiana tabacum | Salicylic acid | |
| CaHDZ13 | TATC-box | TATCCCA | 1904 | 7 | + | Oryza sativa | Gibberellin | |
| CaHDZ13 | ABRE | ACGTG | 809 | 5 | - | Arabidopsis thaliana | Abscisic acid | |
| CaHDZ14 | chs-CMA1a | TTACTTAA | 1860 | 8 | + | Daucus carota | Light |  |
| CaHDZ14 | CGTCA-motif | CGTCA | 1020 | 5 | + | Hordeum vulgare | MeJA |  |
| CaHDZ14 | P-box | CCTTTTG | 15 | 7 | + | Oryza sativa | Gibberellin | |
| CaHDZ14 | Box 4 | ATTAAT | 809 | 6 | + | Petroselinum crispum | Light |  |
| CaHDZ14 | Box 4 | ATTAAT | 882 | 6 | + | Petroselinum crispum | Light |  |
| CaHDZ14 | Box 4 | ATTAAT | 926 | 6 | + | Petroselinum crispum | Light |  |
| CaHDZ14 | Box 4 | ATTAAT | 950 | 6 | + | Petroselinum crispum | Light |  |
| CaHDZ14 | Box 4 | ATTAAT | 1617 | 6 | - | Petroselinum crispum | Light |  |
| CaHDZ14 | TGACG-motif | TGACG | 1020 | 5 | - | Hordeum vulgare | MeJA |  |
| CaHDZ14 | G-Box | CACGTT | 1023 | 6 | + | Pisum sativum | Light |  |
| CaHDZ14 | G-box | CACGTC | 1018 | 6 | + | Zea mays | Light |  |
| CaHDZ14 | G-box | CACGTC | 1569 | 6 | - | Zea mays | Light |  |
| CaHDZ14 | GT1-motif | GGTTAAT | 577 | 7 | + | Avena sativa | Light |  |
| CaHDZ14 | GT1-motif | GGTTAA | 800 | 6 | + | Arabidopsis thaliana | Light |  |
| CaHDZ14 | MBS | CAACTG | 1870 | 6 | - | Arabidopsis thaliana | MYB binding site | |
| CaHDZ14 | GA-motif | ATAGATAA | 199 | 8 | + | Arabidopsis thaliana | Light |  |
| CaHDZ14 | TCA-element | TCAGAAGAGG | 352 | 9 | - | Brassica oleracea | Salicylic acid | |
| CaHDZ14 | TCT-motif | TCTTAC | 978 | 6 | - | Arabidopsis thaliana | Light |  |
| CaHDZ14 | TCT-motif | TCTTAC | 1236 | 6 | - | Arabidopsis thaliana | Light |  |
| CaHDZ14 | TCT-motif | TCTTAC | 1283 | 6 | - | Arabidopsis thaliana | Light |  |
| CaHDZ14 | ABRE | ACGTG | 1018 | 5 | - | Arabidopsis thaliana | Abscisic acid | |
| CaHDZ14 | ABRE | ACGTG | 1023 | 5 | - | Arabidopsis thaliana | Abscisic acid | |
| CaHDZ14 | ABRE | ACGTG | 1570 | 5 | + | Arabidopsis thaliana | Abscisic acid | |
| CaHDZ15 | CGTCA-motif | CGTCA | 293 | 5 | + | Hordeum vulgare | MeJA |  |
| CaHDZ15 | CGTCA-motif | CGTCA | 902 | 5 | + | Hordeum vulgare | MeJA |  |
| CaHDZ15 | CGTCA-motif | CGTCA | 1511 | 5 | - | Hordeum vulgare | MeJA |  |
| CaHDZ15 | GARE-motif | TCTGTTG | 753 | 7 | + | Brassica oleracea | Gibberellin | |
| CaHDZ15 | Box 4 | ATTAAT | 15 | 6 | + | Petroselinum crispum | Light |  |
| CaHDZ15 | Box 4 | ATTAAT | 222 | 6 | + | Petroselinum crispum | Light |  |
| CaHDZ15 | TGACG-motif | TGACG | 293 | 5 | - | Hordeum vulgare | MeJA |  |
| CaHDZ15 | TGACG-motif | TGACG | 902 | 5 | - | Hordeum vulgare | MeJA |  |
| CaHDZ15 | TGACG-motif | TGACG | 1511 | 5 | + | Hordeum vulgare | MeJA |  |
| CaHDZ15 | CCAAT-box | CAACGG | 933 | 6 | + | Hordeum vulgare | MYB binding site | |
| CaHDZ15 | chs-CMA1a | TTACTTAA | 1075 | 8 | - | Daucus carota | Light |  |
| CaHDZ15 | G-Box | CACGTT | 1653 | 6 | - | Pisum sativum | Light |  |
| CaHDZ15 | G-box | TACGTG | 1217 | 6 | - | Arabidopsis thaliana | Light |  |
| CaHDZ15 | GT1-motif | GGTTAAT | 500 | 7 | - | Avena sativa | Light |  |
| CaHDZ15 | GT1-motif | GGTTAA | 501 | 6 | - | Arabidopsis thaliana | Light |  |
| CaHDZ15 | GT1-motif | GGTTAA | 1693 | 6 | + | Arabidopsis thaliana | Light |  |
| CaHDZ15 | MBS | CAACTG | 1298 | 6 | + | Arabidopsis thaliana | MYB binding site | |
| CaHDZ15 | ABRE | ACGTG | 1217 | 5 | - | Arabidopsis thaliana | Abscisic acid | |
| CaHDZ15 | ABRE | ACGTG | 1654 | 5 | + | Arabidopsis thaliana | Abscisic acid | |
| CaHDZ16 | CGTCA-motif | CGTCA | 319 | 5 | - | Hordeum vulgare | MeJA |  |
| CaHDZ16 | CGTCA-motif | CGTCA | 389 | 5 | + | Hordeum vulgare | MeJA |  |
| CaHDZ16 | CGTCA-motif | CGTCA | 399 | 5 | - | Hordeum vulgare | MeJA |  |
| CaHDZ16 | CGTCA-motif | CGTCA | 527 | 5 | + | Hordeum vulgare | MeJA |  |
| CaHDZ16 | CGTCA-motif | CGTCA | 1919 | 5 | - | Hordeum vulgare | MeJA |  |
| CaHDZ16 | Gap-box | CAAATGAA(A/G)A | 1046 | 9.5 | - | Arabidopsis thaliana | Light |  |
| CaHDZ16 | MBSI | aaaAaaC(G/C)GTTA | 469 | 10.5 | - | Petunia hybrida | MYB binding site | |
| CaHDZ16 | MBSI | aaaAaaC(G/C)GTTA | 501 | 10.5 | - | Petunia hybrida | MYB binding site | |
| CaHDZ16 | P-box | CCTTTTG | 1777 | 7 | + | Oryza sativa | Gibberellin | |
| CaHDZ16 | AE-box | AGAAACAA | 1401 | 8 | - | Arabidopsis thaliana | Light |  |
| CaHDZ16 | AE-box | AGAAACTT | 1902 | 8 | - | Arabidopsis thaliana | Light |  |
| CaHDZ16 | Box 4 | ATTAAT | 242 | 6 | + | Petroselinum crispum | Light |  |
| CaHDZ16 | Box 4 | ATTAAT | 588 | 6 | + | Petroselinum crispum | Light |  |
| CaHDZ16 | Box 4 | ATTAAT | 1062 | 6 | - | Petroselinum crispum | Light |  |
| CaHDZ16 | Box 4 | ATTAAT | 1097 | 6 | - | Petroselinum crispum | Light |  |
| CaHDZ16 | Box 4 | ATTAAT | 1105 | 6 | - | Petroselinum crispum | Light |  |
| CaHDZ16 | TGACG-motif | TGACG | 319 | 5 | + | Hordeum vulgare | MeJA |  |
| CaHDZ16 | TGACG-motif | TGACG | 389 | 5 | - | Hordeum vulgare | MeJA |  |
| CaHDZ16 | TGACG-motif | TGACG | 399 | 5 | + | Hordeum vulgare | MeJA |  |
| CaHDZ16 | TGACG-motif | TGACG | 527 | 5 | - | Hordeum vulgare | MeJA |  |
| CaHDZ16 | TGACG-motif | TGACG | 1919 | 5 | + | Hordeum vulgare | MeJA |  |
| CaHDZ16 | TGA-box | TGACGTAA | 399 | 8 | + | Glycine max | Auxin |  |
| CaHDZ16 | GATA-motif | AAGATAAGATT | 113 | 10 | + | Arabidopsis thaliana | Light |  |
| CaHDZ16 | G-box | CACGAC | 610 | 6 | + | Zea mays | Light |  |
| CaHDZ16 | G-box | CACGTC | 950 | 6 | - | Zea mays | Light |  |
| CaHDZ16 | GT1-motif | GGTTAA | 435 | 6 | - | Arabidopsis thaliana | Light |  |
| CaHDZ16 | ARE | AAACCA | 890 | 6 | - | Zea mays | Anaerobic induction | |
| CaHDZ16 | ARE | AAACCA | 1444 | 6 | - | Zea mays | Anaerobic induction | |
| CaHDZ16 | LTR | CCGAAA | 773 | 6 | + | Hordeum vulgare | Low temperature | |
| CaHDZ16 | ABRE | TACGGTC | 687 | 7 | - | Arabidopsis thaliana | Abscisic acid | |
| CaHDZ16 | ABRE | ACGTG | 951 | 5 | + | Arabidopsis thaliana | Abscisic acid | |
| CaHDZ17 | ACE | CTAACGTATT | 1774 | 9 | - | Petroselinum crispum | Light |  |
| CaHDZ17 | CGTCA-motif | CGTCA | 741 | 5 | - | Hordeum vulgare | MeJA |  |
| CaHDZ17 | 3-AF1 binding site | TAAGAGAGGAA | 890 | 10 | + | Solanum tuberosum | Light |  |
| CaHDZ17 | TGACG-motif | TGACG | 741 | 5 | + | Hordeum vulgare | MeJA |  |
| CaHDZ17 | G-Box | CACGTT | 376 | 6 | + | Pisum sativum | Light |  |
| CaHDZ17 | G-Box | CACGTT | 716 | 6 | + | Pisum sativum | Light |  |
| CaHDZ17 | G-Box | CACGTG | 1299 | 6 | - | Pisum sativum | Light |  |
| CaHDZ17 | G-box | ACACGTGT | 1298 | 8 | - | Brassica napus | Light |  |
| CaHDZ17 | G-box | CACGTG | 1299 | 6 | - | Arabidopsis thaliana | Light |  |
| CaHDZ17 | G-box | CACGAC | 1793 | 6 | + | Zea mays | Light |  |
| CaHDZ17 | GT1-motif | GGTTAAT | 42 | 7 | + | Avena sativa | Light |  |
| CaHDZ17 | ARE | AAACCA | 998 | 6 | + | Zea mays | Anaerobic induction | |
| CaHDZ17 | ARE | AAACCA | 1829 | 6 | + | Zea mays | Anaerobic induction | |
| CaHDZ17 | MBS | CAACTG | 1488 | 6 | + | Arabidopsis thaliana | MYB binding site | |
| CaHDZ17 | MBS | CAACTG | 1560 | 6 | + | Arabidopsis thaliana | MYB binding site | |
| CaHDZ17 | TCA-element | TCAGAAGAGG | 1248 | 9 | - | Brassica oleracea | Salicylic acid | |
| CaHDZ17 | TCT-motif | TCTTAC | 488 | 6 | - | Arabidopsis thaliana | Light |  |
| CaHDZ17 | TCT-motif | TCTTAC | 1179 | 6 | - | Arabidopsis thaliana | Light |  |
| CaHDZ17 | ABRE | ACGTG | 376 | 5 | - | Arabidopsis thaliana | Abscisic acid | |
| CaHDZ17 | ABRE | ACGTG | 716 | 5 | - | Arabidopsis thaliana | Abscisic acid | |
| CaHDZ17 | ABRE | CACGTG | 1299 | 6 | - | Arabidopsis thaliana | Abscisic acid | |
| CaHDZ17 | ABRE | ACGTG | 1300 | 5 | + | Arabidopsis thaliana | Abscisic acid | |
| CaHDZ18 | CGTCA-motif | CGTCA | 183 | 5 | - | Hordeum vulgare | MeJA |  |
| CaHDZ18 | 3-AF1 binding site | TAAGAGAGGAA | 1469 | 11 | - | Solanum tuberosum | Light |  |
| CaHDZ18 | AE-box | AGAAACAA | 1868 | 8 | - | Arabidopsis thaliana | Light |  |
| CaHDZ18 | Box 4 | ATTAAT | 350 | 6 | + | Petroselinum crispum | Light |  |
| CaHDZ18 | Box 4 | ATTAAT | 1203 | 6 | - | Petroselinum crispum | Light |  |
| CaHDZ18 | Box 4 | ATTAAT | 1510 | 6 | - | Petroselinum crispum | Light |  |
| CaHDZ18 | Box 4 | ATTAAT | 1694 | 6 | - | Petroselinum crispum | Light |  |
| CaHDZ18 | Box 4 | ATTAAT | 1748 | 6 | - | Petroselinum crispum | Light |  |
| CaHDZ18 | TCCC-motif | TCTCCCT | 1672 | 7 | + | Spinacia oleracea | Light |  |
| CaHDZ18 | TGACG-motif | TGACG | 183 | 5 | + | Hordeum vulgare | MeJA |  |
| CaHDZ18 | CCAAT-box | CAACGG | 1605 | 6 | - | Hordeum vulgare | MYB binding site | |
| CaHDZ18 | chs-CMA1a | TTACTTAA | 1332 | 8 | - | Daucus carota | Light |  |
| CaHDZ18 | WUN-motif | AAATTTCCT | 1465 | 9 | + | Brassica oleracea | Wound |  |
| CaHDZ18 | GATA-motif | GATAGGG | 687 | 7 | + | Pisum sativum | Light |  |
| CaHDZ18 | GT1-motif | GGTTAA | 593 | 6 | + | Arabidopsis thaliana | Light |  |
| CaHDZ18 | TCA-element | CCATCTTTTT | 1222 | 9 | + | Nicotiana tabacum | Salicylic acid | |
| CaHDZ18 | TCT-motif | TCTTAC | 1475 | 6 | + | Arabidopsis thaliana | Light |  |
| CaHDZ18 | ATCT-motif | AATCTAATCC | 192 | 9 | + | Pisum sativum | Light |  |
| CaHDZ19 | CGTCA-motif | CGTCA | 647 | 5 | - | Hordeum vulgare | MeJA |  |
| CaHDZ19 | CGTCA-motif | CGTCA | 933 | 5 | - | Hordeum vulgare | MeJA |  |
| CaHDZ19 | AE-box | AGAAACAA | 562 | 8 | - | Arabidopsis thaliana | Light |  |
| CaHDZ19 | GARE-motif | TCTGTTG | 1539 | 7 | + | Brassica oleracea | Gibberellin | |
| CaHDZ19 | Box 4 | ATTAAT | 830 | 6 | + | Petroselinum crispum | Light |  |
| CaHDZ19 | AAAC-motif | CAATCAAAACCT | 1557 | 11 | + | Spinacia oleracea | Light |  |
| CaHDZ19 | TGACG-motif | TGACG | 647 | 5 | + | Hordeum vulgare | MeJA |  |
| CaHDZ19 | TGACG-motif | TGACG | 933 | 5 | + | Hordeum vulgare | MeJA |  |
| CaHDZ19 | GTGGC-motif | CAGCGTGTGGC | 1808 | 10 | - | Hordeum vulgare | Light |  |
| CaHDZ19 | CCAAT-box | CAACGG | 883 | 6 | - | Hordeum vulgare | MYB binding site | |
| CaHDZ19 | TC-rich repeats | GTTTTCTTAC | 354 | 9 | + | Nicotiana tabacum | Defense and stress | |
| CaHDZ19 | GT1-motif | GGTTAA | 187 | 6 | + | Arabidopsis thaliana | Light |  |
| CaHDZ19 | GT1-motif | GGTTAA | 1218 | 6 | - | Arabidopsis thaliana | Light |  |
| CaHDZ19 | GT1-motif | GGTTAA | 1253 | 6 | - | Arabidopsis thaliana | Light |  |
| CaHDZ19 | GT1-motif | GGTTAA | 1486 | 6 | + | Arabidopsis thaliana | Light |  |
| CaHDZ19 | GT1-motif | GGTTAA | 1488 | 6 | - | Arabidopsis thaliana | Light |  |
| CaHDZ19 | MRE | AACCTAA | 1483 | 7 | - | Petroselinum crispum | MYB binding site | |
| CaHDZ19 | ARE | AAACCA | 670 | 6 | + | Zea mays | Anaerobic induction | |
| CaHDZ19 | ARE | AAACCA | 685 | 6 | - | Zea mays | Anaerobic induction | |
| CaHDZ19 | ARE | AAACCA | 1206 | 6 | + | Zea mays | Anaerobic induction | |
| CaHDZ19 | ARE | AAACCA | 1240 | 6 | + | Zea mays | Anaerobic induction | |
| CaHDZ19 | LTR | CCGAAA | 403 | 6 | + | Hordeum vulgare | Low temperature | |
| CaHDZ19 | LTR | CCGAAA | 639 | 6 | - | Hordeum vulgare | Low temperature | |
| CaHDZ19 | LTR | CCGAAA | 1171 | 6 | - | Hordeum vulgare | Low temperature | |
| CaHDZ19 | MBS | CAACTG | 955 | 6 | + | Arabidopsis thaliana | MYB binding site | |
| CaHDZ19 | TCT-motif | TCTTAC | 358 | 6 | + | Arabidopsis thaliana | Light |  |
| CaHDZ19 | TCT-motif | TCTTAC | 567 | 6 | + | Arabidopsis thaliana | Light |  |
| CaHDZ19 | TGA-element | AACGAC | 947 | 6 | + | Brassica oleracea | Auxin |  |
| CaHDZ19 | TGA-element | AACGAC | 960 | 6 | - | Brassica oleracea | Auxin |  |
| CaHDZ19 | TGA-element | AACGAC | 1006 | 6 | - | Brassica oleracea | Auxin |  |
| CaHDZ20 | CGTCA-motif | CGTCA | 197 | 5 | - | Hordeum vulgare | MeJA |  |
| CaHDZ20 | CGTCA-motif | CGTCA | 334 | 5 | - | Hordeum vulgare | MeJA |  |
| CaHDZ20 | CGTCA-motif | CGTCA | 791 | 5 | + | Hordeum vulgare | MeJA |  |
| CaHDZ20 | CGTCA-motif | CGTCA | 910 | 5 | + | Hordeum vulgare | MeJA |  |
| CaHDZ20 | CGTCA-motif | CGTCA | 1693 | 5 | - | Hordeum vulgare | MeJA |  |
| CaHDZ20 | CGTCA-motif | CGTCA | 1772 | 5 | + | Hordeum vulgare | MeJA |  |
| CaHDZ20 | Gap-box | CAAATGAA(A/G)A | 1645 | 9.5 | + | Arabidopsis thaliana | Light |  |
| CaHDZ20 | 3-AF1 binding site | TAAGAGAGGAA | 804 | 10 | + | Solanum tuberosum | Light |  |
| CaHDZ20 | TGACG-motif | TGACG | 197 | 5 | + | Hordeum vulgare | MeJA |  |
| CaHDZ20 | TGACG-motif | TGACG | 334 | 5 | + | Hordeum vulgare | MeJA |  |
| CaHDZ20 | TGACG-motif | TGACG | 791 | 5 | - | Hordeum vulgare | MeJA |  |
| CaHDZ20 | TGACG-motif | TGACG | 910 | 5 | - | Hordeum vulgare | MeJA |  |
| CaHDZ20 | TGACG-motif | TGACG | 1693 | 5 | + | Hordeum vulgare | MeJA |  |
| CaHDZ20 | TGACG-motif | TGACG | 1772 | 5 | - | Hordeum vulgare | MeJA |  |
| CaHDZ20 | AT1-motif | AATTATTTTTTATT | 489 | 13 | - | Solanum tuberosum | Light |  |
| CaHDZ20 | AT1-motif | AATTATTTTTTATT | 633 | 13 | - | Solanum tuberosum | Light |  |
| CaHDZ20 | L-box | ATCCCACCTAC | 241 | 10 | - | Petroselinum crispum | Light |  |
| CaHDZ20 | G-Box | CACGTT | 1519 | 6 | - | Pisum sativum | Light |  |
| CaHDZ20 | GATA-motif | AAGATAAGATT | 1397 | 10 | - | Arabidopsis thaliana | Light |  |
| CaHDZ20 | G-box | TACGTG | 1019 | 6 | + | Arabidopsis thaliana | Light |  |
| CaHDZ20 | G-box | TACGTG | 1058 | 6 | + | Arabidopsis thaliana | Light |  |
| CaHDZ20 | GT1-motif | GGTTAA | 1679 | 6 | + | Arabidopsis thaliana | Light |  |
| CaHDZ20 | ARE | AAACCA | 433 | 6 | - | Zea mays | Anaerobic induction | |
| CaHDZ20 | ARE | AAACCA | 823 | 6 | - | Zea mays | Anaerobic induction | |
| CaHDZ20 | ARE | AAACCA | 1462 | 6 | - | Zea mays | Anaerobic induction | |
| CaHDZ20 | LTR | CCGAAA | 735 | 6 | - | Hordeum vulgare | Low temperature | |
| CaHDZ20 | MBS | CAACTG | 1246 | 6 | - | Arabidopsis thaliana | MYB binding site | |
| CaHDZ20 | MBS | CAACTG | 1468 | 6 | + | Arabidopsis thaliana | MYB binding site | |
| CaHDZ20 | GA-motif | ATAGATAA | 1332 | 8 | + | Arabidopsis thaliana | Light |  |
| CaHDZ20 | ABRE | ACGTG | 1020 | 5 | + | Arabidopsis thaliana | Abscisic acid | |
| CaHDZ20 | ABRE | ACGTG | 1059 | 5 | + | Arabidopsis thaliana | Abscisic acid | |
| CaHDZ20 | ABRE | ACGTG | 1520 | 5 | + | Arabidopsis thaliana | Abscisic acid | |
| CaHDZ21 | ACE | GACACGTATG | 1414 | 9 | - | Petroselinum crispum | Light |  |
| CaHDZ21 | CGTCA-motif | CGTCA | 1768 | 5 | - | Hordeum vulgare | MeJA |  |
| CaHDZ21 | AE-box | AGAAACAA | 701 | 8 | - | Arabidopsis thaliana | Light |  |
| CaHDZ21 | AE-box | AGAAACAA | 1746 | 8 | + | Arabidopsis thaliana | Light |  |
| CaHDZ21 | Box 4 | ATTAAT | 210 | 6 | + | Petroselinum crispum | Light |  |
| CaHDZ21 | TCCC-motif | TCTCCCT | 1702 | 7 | - | Spinacia oleracea | Light |  |
| CaHDZ21 | TGACG-motif | TGACG | 1768 | 5 | + | Hordeum vulgare | MeJA |  |
| CaHDZ21 | TC-rich repeats | GTTTTCTTAC | 1977 | 9 | + | Nicotiana tabacum | Defense and stress | |
| CaHDZ21 | GATA-motif | AAGATAAGATT | 20 | 10 | - | Arabidopsis thaliana | Light |  |
| CaHDZ21 | GATA-motif | GATAGGA | 153 | 7 | - | Arabidopsis thaliana | Light |  |
| CaHDZ21 | GATA-motif | GATAGGA | 678 | 7 | - | Arabidopsis thaliana | Light |  |
| CaHDZ21 | GT1-motif | GGTTAA | 1354 | 6 | - | Arabidopsis thaliana | Light |  |
| CaHDZ21 | LTR | CCGAAA | 797 | 6 | + | Hordeum vulgare | Low temperature | |
| CaHDZ21 | LTR | CCGAAA | 1169 | 6 | + | Hordeum vulgare | Low temperature | |
| CaHDZ21 | MBS | CAACTG | 226 | 6 | - | Arabidopsis thaliana | MYB binding site | |
| CaHDZ21 | MBS | CAACTG | 528 | 6 | + | Arabidopsis thaliana | MYB binding site | |
| CaHDZ21 | ABRE | CGCACGTGTC | 670 | 9 | + | Hordeum vulgare | Abscisic acid | |
| CaHDZ23 | ACE | CTAACGTATT | 229 | 9 | - | Petroselinum crispum | Light |  |
| CaHDZ23 | ACE | CTAACGTATT | 246 | 9 | - | Petroselinum crispum | Light |  |
| CaHDZ23 | ACE | CTAACGTATT | 262 | 9 | - | Petroselinum crispum | Light |  |
| CaHDZ23 | CGTCA-motif | CGTCA | 636 | 5 | + | Hordeum vulgare | MeJA |  |
| CaHDZ23 | AE-box | AGAAACAA | 767 | 8 | + | Arabidopsis thaliana | Light |  |
| CaHDZ23 | AE-box | AGAAACTT | 1261 | 8 | - | Arabidopsis thaliana | Light |  |
| CaHDZ23 | I-box | atGATAAGGTC | 629 | 10 | + | Helianthus annuus | Light |  |
| CaHDZ23 | I-box | GTATAAGGCC | 993 | 9 | - | Larix laricina | Light |  |
| CaHDZ23 | Box 4 | ATTAAT | 1754 | 6 | - | Petroselinum crispum | Light |  |
| CaHDZ23 | TGACG-motif | TGACG | 636 | 5 | - | Hordeum vulgare | MeJA |  |
| CaHDZ23 | GT1-motif | GGTTAAT | 1705 | 7 | + | Avena sativa | Light |  |
| CaHDZ23 | MRE | AACCTAA | 194 | 7 | - | Petroselinum crispum | MYB binding site | |
| CaHDZ23 | ARE | AAACCA | 143 | 6 | + | Zea mays | Anaerobic induction | |
| CaHDZ23 | ARE | AAACCA | 1597 | 6 | + | Zea mays | Anaerobic induction | |
| CaHDZ23 | ARE | AAACCA | 1962 | 6 | + | Zea mays | Anaerobic induction | |
| CaHDZ23 | LTR | CCGAAA | 140 | 6 | + | Hordeum vulgare | Low temperature | |
| CaHDZ23 | MBS | CAACTG | 1370 | 6 | - | Arabidopsis thaliana | MYB binding site | |
| CaHDZ23 | MBS | CAACTG | 1389 | 6 | + | Arabidopsis thaliana | MYB binding site | |
| CaHDZ23 | GA-motif | ATAGATAA | 664 | 8 | - | Arabidopsis thaliana | Light |  |
| CaHDZ25 | 3-AF1 binding site | TAAGAGAGGAA | 281 | 10 | - | Solanum tuberosum | Light |  |
| CaHDZ25 | TC-rich repeats | GTTTTCTTAC | 369 | 9 | + | Nicotiana tabacum | Defense and stress | |
| CaHDZ25 | I-box | cCATATCCAAT | 1400 | 11 | + | Flaveria trinervia | Light |  |
| CaHDZ25 | Box 4 | ATTAAT | 317 | 6 | + | Petroselinum crispum | Light |  |
| CaHDZ25 | Box 4 | ATTAAT | 409 | 6 | + | Petroselinum crispum | Light |  |
| CaHDZ25 | Box 4 | ATTAAT | 639 | 6 | + | Petroselinum crispum | Light |  |
| CaHDZ25 | Box 4 | ATTAAT | 686 | 6 | + | Petroselinum crispum | Light |  |
| CaHDZ25 | Box 4 | ATTAAT | 758 | 6 | + | Petroselinum crispum | Light |  |
| CaHDZ25 | Box 4 | ATTAAT | 1084 | 6 | - | Petroselinum crispum | Light |  |
| CaHDZ25 | Box 4 | ATTAAT | 1088 | 6 | - | Petroselinum crispum | Light |  |
| CaHDZ25 | Box 4 | ATTAAT | 1505 | 6 | - | Petroselinum crispum | Light |  |
| CaHDZ25 | Box 4 | ATTAAT | 1519 | 6 | - | Petroselinum crispum | Light |  |
| CaHDZ25 | G-Box | CACGTT | 1359 | 6 | - | Pisum sativum | Light |  |
| CaHDZ25 | GATA-motif | AAGATAAGATT | 646 | 10 | + | Arabidopsis thaliana | Light |  |
| CaHDZ25 | GATA-motif | AAGATAAGATT | 1166 | 10 | - | Arabidopsis thaliana | Light |  |
| CaHDZ25 | G-box | CACGAC | 1686 | 6 | + | Zea mays | Light |  |
| CaHDZ25 | ARE | AAACCA | 139 | 6 | - | Zea mays | Anaerobic induction | |
| CaHDZ25 | ARE | AAACCA | 234 | 6 | - | Zea mays | Anaerobic induction | |
| CaHDZ25 | ARE | AAACCA | 292 | 6 | + | Zea mays | Anaerobic induction | |
| CaHDZ25 | ARE | AAACCA | 976 | 6 | - | Zea mays | Anaerobic induction | |
| CaHDZ25 | ARE | AAACCA | 1692 | 6 | + | Zea mays | Anaerobic induction | |
| CaHDZ25 | ARE | AAACCA | 1958 | 6 | + | Zea mays | Anaerobic induction | |
| CaHDZ25 | TCA-element | CCATCTTTTT | 1036 | 9 | - | Nicotiana tabacum | Salicylic acid | |
| CaHDZ25 | chs-CMA2a | TCACTTGA | 850 | 8 | + | Petroselinum crispum | Light |  |
| CaHDZ25 | TCT-motif | TCTTAC | 373 | 6 | + | Arabidopsis thaliana | Light |  |
| CaHDZ25 | TCT-motif | TCTTAC | 1173 | 6 | + | Arabidopsis thaliana | Light |  |
| CaHDZ25 | TCT-motif | TCTTAC | 1566 | 6 | + | Arabidopsis thaliana | Light |  |
| CaHDZ25 | ABRE | ACGTG | 1360 | 5 | + | Arabidopsis thaliana | Abscisic acid | |
| CaHDZ25 | ATCT-motif | AATCTAATCC | 1166 | 9 | + | Pisum sativum | Light |  |
| CaHDZ26 | CGTCA-motif | CGTCA | 28 | 5 | - | Hordeum vulgare | MeJA |  |
| CaHDZ26 | CGTCA-motif | CGTCA | 776 | 5 | + | Hordeum vulgare | MeJA |  |
| CaHDZ26 | CGTCA-motif | CGTCA | 1227 | 5 | - | Hordeum vulgare | MeJA |  |
| CaHDZ26 | CGTCA-motif | CGTCA | 1767 | 5 | - | Hordeum vulgare | MeJA |  |
| CaHDZ26 | TC-rich repeats | ATTCTCTAAC | 623 | 9 | + | Nicotiana tabacum | Defense and stress | |
| CaHDZ26 | P-box | CCTTTTG | 1760 | 7 | - | Oryza sativa | Gibberellin | |
| CaHDZ26 | Box 4 | ATTAAT | 1699 | 6 | - | Petroselinum crispum | Light |  |
| CaHDZ26 | Box 4 | ATTAAT | 1928 | 6 | - | Petroselinum crispum | Light |  |
| CaHDZ26 | TCCC-motif | TCTCCCT | 197 | 7 | - | Spinacia oleracea | Light |  |
| CaHDZ26 | TGACG-motif | TGACG | 28 | 5 | + | Hordeum vulgare | MeJA |  |
| CaHDZ26 | TGACG-motif | TGACG | 776 | 5 | - | Hordeum vulgare | MeJA |  |
| CaHDZ26 | TGACG-motif | TGACG | 1227 | 5 | + | Hordeum vulgare | MeJA |  |
| CaHDZ26 | TGACG-motif | TGACG | 1767 | 5 | + | Hordeum vulgare | MeJA |  |
| CaHDZ26 | ARE | AAACCA | 790 | 6 | + | Zea mays | Anaerobic induction | |
| CaHDZ26 | ARE | AAACCA | 833 | 6 | + | Zea mays | Anaerobic induction | |
| CaHDZ26 | ARE | AAACCA | 933 | 6 | - | Zea mays | Anaerobic induction | |
| CaHDZ26 | ARE | AAACCA | 1003 | 6 | + | Zea mays | Anaerobic induction | |
| CaHDZ26 | ARE | AAACCA | 1473 | 6 | - | Zea mays | Anaerobic induction | |
| CaHDZ26 | TATC-box | TATCCCA | 1945 | 7 | + | Oryza sativa | Gibberellin | |
| CaHDZ26 | ABRE | GCAACGTGTC | 1570 | 9 | + | Hordeum vulgare | Abscisic acid | |
| CaHDZ27 | CGTCA-motif | CGTCA | 746 | 5 | + | Hordeum vulgare | MeJA |  |
| CaHDZ27 | AE-box | AGAAACAA | 39 | 8 | - | Arabidopsis thaliana | Light |  |
| CaHDZ27 | GARE-motif | TCTGTTG | 1969 | 7 | - | Brassica oleracea | Gibberellin | |
| CaHDZ27 | Box 4 | ATTAAT | 1358 | 6 | - | Petroselinum crispum | Light |  |
| CaHDZ27 | Box 4 | ATTAAT | 1372 | 6 | - | Petroselinum crispum | Light |  |
| CaHDZ27 | Box 4 | ATTAAT | 1376 | 6 | - | Petroselinum crispum | Light |  |
| CaHDZ27 | Box 4 | ATTAAT | 1408 | 6 | - | Petroselinum crispum | Light |  |
| CaHDZ27 | Box 4 | ATTAAT | 1992 | 6 | - | Petroselinum crispum | Light |  |
| CaHDZ27 | TGACG-motif | TGACG | 746 | 5 | - | Hordeum vulgare | MeJA |  |
| CaHDZ27 | GATA-motif | GATAGGG | 1291 | 7 | - | Pisum sativum | Light |  |
| CaHDZ27 | G-box | TACGTG | 283 | 6 | - | Arabidopsis thaliana | Light |  |
| CaHDZ27 | Box II | TGGTAATAA | 1209 | 9 | + | Solanum tuberosum | Light |  |
| CaHDZ27 | ARE | AAACCA | 165 | 6 | + | Zea mays | Anaerobic induction | |
| CaHDZ27 | ARE | AAACCA | 461 | 6 | - | Zea mays | Anaerobic induction | |
| CaHDZ27 | LTR | CCGAAA | 317 | 6 | - | Hordeum vulgare | Low temperature | |
| CaHDZ27 | ABRE | ACGTG | 283 | 5 | - | Arabidopsis thaliana | Abscisic acid | |
| CaHDZ28 | Box 4 | ATTAAT | 214 | 6 | + | Petroselinum crispum | Light |  |
| CaHDZ28 | Box 4 | ATTAAT | 635 | 6 | + | Petroselinum crispum | Light |  |
| CaHDZ28 | Box 4 | ATTAAT | 1255 | 6 | - | Petroselinum crispum | Light |  |
| CaHDZ28 | Box 4 | ATTAAT | 1292 | 6 | - | Petroselinum crispum | Light |  |
| CaHDZ28 | Box 4 | ATTAAT | 1678 | 6 | - | Petroselinum crispum | Light |  |
| CaHDZ28 | TATC-box | TATCCCA | 710 | 7 | + | Oryza sativa | Gibberellin | |
| CaHDZ28 | Sp1 | GGGCGG | 115 | 6 | - | Oryza sativa | Light |  |
| CaHDZ28 | TC-rich repeats | ATTCTCTAAC | 1577 | 9 | + | Nicotiana tabacum | Defense and stress | |
| CaHDZ28 | ATC-motif | AGTAATCT | 236 | 8 | - | Spinacia oleracea | Light |  |
| CaHDZ28 | G-Box | CACGTG | 861 | 6 | + | Pisum sativum | Light |  |
| CaHDZ28 | G-box | GCCACGTGGA | 859 | 9 | + | Arabidopsis thaliana | Light |  |
| CaHDZ28 | G-box | CACGTG | 861 | 6 | + | Arabidopsis thaliana | Light |  |
| CaHDZ28 | GT1-motif | GGTTAAT | 1191 | 7 | - | Avena sativa | Light |  |
| CaHDZ28 | GT1-motif | GGTTAA | 1192 | 6 | - | Arabidopsis thaliana | Light |  |
| CaHDZ28 | GT1-motif | GGTTAAT | 1755 | 7 | - | Avena sativa | Light |  |
| CaHDZ28 | GT1-motif | GGTTAA | 1756 | 6 | - | Arabidopsis thaliana | Light |  |
| CaHDZ28 | ARE | AAACCA | 1913 | 6 | + | Zea mays | Anaerobic induction | |
| CaHDZ28 | chs-CMA2a | TCACTTGA | 988 | 8 | + | Petroselinum crispum | Light |  |
| CaHDZ28 | ABRE | CACGTG | 861 | 6 | + | Arabidopsis thaliana | Abscisic acid | |
| CaHDZ28 | ABRE | ACGTG | 862 | 5 | + | Arabidopsis thaliana | Abscisic acid | |
| CaHDZ31 | TC-rich repeats | ATTCTCTAAC | 1010 | 9 | + | Nicotiana tabacum | Defense and stress | |
| CaHDZ31 | Box 4 | ATTAAT | 556 | 6 | + | Petroselinum crispum | Light |  |
| CaHDZ31 | Box 4 | ATTAAT | 1363 | 6 | - | Petroselinum crispum | Light |  |
| CaHDZ31 | Box 4 | ATTAAT | 1703 | 6 | - | Petroselinum crispum | Light |  |
| CaHDZ31 | Box 4 | ATTAAT | 1971 | 6 | - | Petroselinum crispum | Light |  |
| CaHDZ31 | G-box | TAACACGTAG | 1140 | 9 | - | Brassica oleracea | Light |  |
| CaHDZ31 | G-box | TAACACGTAG | 1720 | 9 | + | Brassica oleracea | Light |  |
| CaHDZ31 | MBS | CAACTG | 1298 | 6 | - | Arabidopsis thaliana | MYB binding site | |
| CaHDZ32 | chs-CMA1a | TTACTTAA | 170 | 8 | - | Daucus carota | Light |  |
| CaHDZ32 | CGTCA-motif | CGTCA | 1959 | 5 | + | Hordeum vulgare | MeJA |  |
| CaHDZ32 | AT1-motif | AATTATTTTTTATT | 6 | 13 | - | Solanum tuberosum | Light |  |
| CaHDZ32 | P-box | CCTTTTG | 643 | 7 | + | Oryza sativa | Gibberellin | |
| CaHDZ32 | 4cl-CMA2b | TCTCACCAACCACA | 281 | 13 | + | Solanum tuberosum | Light |  |
| CaHDZ32 | Box 4 | ATTAAT | 197 | 6 | + | Petroselinum crispum | Light |  |
| CaHDZ32 | Box 4 | ATTAAT | 1963 | 6 | - | Petroselinum crispum | Light |  |
| CaHDZ32 | TGACG-motif | TGACG | 1959 | 5 | - | Hordeum vulgare | MeJA |  |
| CaHDZ32 | GATA-motif | AAGATAAGATT | 1622 | 10 | + | Arabidopsis thaliana | Light |  |
| CaHDZ32 | GT1-motif | GGTTAA | 664 | 6 | - | Arabidopsis thaliana | Light |  |
| CaHDZ32 | GT1-motif | GGTTAA | 761 | 6 | - | Arabidopsis thaliana | Light |  |
| CaHDZ32 | ARE | AAACCA | 1165 | 6 | + | Zea mays | Anaerobic induction | |
| CaHDZ32 | TCA-element | CCATCTTTTT | 946 | 9 | + | Nicotiana tabacum | Salicylic acid | |
| CaHDZ32 | TCT-motif | TCTTAC | 1267 | 6 | + | Arabidopsis thaliana | Light |  |
| CaHDZ32 | TGA-element | AACGAC | 746 | 6 | + | Brassica oleracea | Auxin |  |
| CaHDZ33 | CGTCA-motif | CGTCA | 776 | 5 | - | Hordeum vulgare | MeJA |  |
| CaHDZ33 | CGTCA-motif | CGTCA | 779 | 5 | + | Hordeum vulgare | MeJA |  |
| CaHDZ33 | CGTCA-motif | CGTCA | 1203 | 5 | + | Hordeum vulgare | MeJA |  |
| CaHDZ33 | CGTCA-motif | CGTCA | 1429 | 5 | - | Hordeum vulgare | MeJA |  |
| CaHDZ33 | Box 4 | ATTAAT | 610 | 6 | + | Petroselinum crispum | Light |  |
| CaHDZ33 | Box 4 | ATTAAT | 614 | 6 | + | Petroselinum crispum | Light |  |
| CaHDZ33 | TGACG-motif | TGACG | 776 | 5 | + | Hordeum vulgare | MeJA |  |
| CaHDZ33 | TGACG-motif | TGACG | 779 | 5 | - | Hordeum vulgare | MeJA |  |
| CaHDZ33 | TGACG-motif | TGACG | 1203 | 5 | - | Hordeum vulgare | MeJA |  |
| CaHDZ33 | TGACG-motif | TGACG | 1429 | 5 | + | Hordeum vulgare | MeJA |  |
| CaHDZ33 | AuxRR-core | GGTCCAT | 172 | 7 | - | Nicotiana tabacum | Auxin |  |
| CaHDZ33 | Sp1 | GGGCGG | 1167 | 6 | - | Oryza sativa | Light |  |
| CaHDZ33 | TC-rich repeats | GTTTTCTTAC | 1401 | 9 | + | Nicotiana tabacum | Defense and stress | |
| CaHDZ33 | G-Box | CACGTG | 1104 | 6 | - | Pisum sativum | Light |  |
| CaHDZ33 | GATA-motif | GATAGGA | 1900 | 7 | + | Arabidopsis thaliana | Light |  |
| CaHDZ33 | G-box | CACGTG | 1104 | 6 | - | Arabidopsis thaliana | Light |  |
| CaHDZ33 | G-box | CACGAC | 1450 | 6 | - | Zea mays | Light |  |
| CaHDZ33 | GT1-motif | GGTTAA | 944 | 6 | - | Arabidopsis thaliana | Light |  |
| CaHDZ33 | GT1-motif | GGTTAA | 1620 | 6 | + | Arabidopsis thaliana | Light |  |
| CaHDZ33 | GT1-motif | GGTTAA | 1708 | 6 | - | Arabidopsis thaliana | Light |  |
| CaHDZ33 | ARE | AAACCA | 1313 | 6 | - | Zea mays | Anaerobic induction | |
| CaHDZ33 | ARE | AAACCA | 1539 | 6 | - | Zea mays | Anaerobic induction | |
| CaHDZ33 | ARE | AAACCA | 1838 | 6 | + | Zea mays | Anaerobic induction | |
| CaHDZ33 | GA-motif | ATAGATAA | 499 | 8 | + | Arabidopsis thaliana | Light |  |
| CaHDZ33 | GA-motif | ATAGATAA | 1186 | 8 | - | Arabidopsis thaliana | Light |  |
| CaHDZ33 | TCA-element | TCAGAAGAGG | 17 | 9 | - | Brassica oleracea | Salicylic acid | |
| CaHDZ33 | TCA-element | CCATCTTTTT | 1172 | 9 | + | Nicotiana tabacum | Salicylic acid | |
| CaHDZ33 | TCA-element | CCATCTTTTT | 1959 | 9 | + | Nicotiana tabacum | Salicylic acid | |
| CaHDZ33 | TCT-motif | TCTTAC | 22 | 6 | + | Arabidopsis thaliana | Light |  |
| CaHDZ33 | ABRE | CACGTG | 1104 | 6 | - | Arabidopsis thaliana | Abscisic acid | |
| CaHDZ33 | ABRE | ACGTG | 1105 | 5 | + | Arabidopsis thaliana | Abscisic acid | |
| CaHDZ34 | 3-AF1 binding site | TAAGAGAGGAA | 1809 | 10 | - | Solanum tuberosum | Light |  |
| CaHDZ34 | GT1-motif | GGTTAA | 64 | 6 | + | Arabidopsis thaliana | Light |  |
| CaHDZ34 | GT1-motif | GGTTAA | 545 | 6 | + | Arabidopsis thaliana | Light |  |
| CaHDZ34 | GT1-motif | GGTTAA | 909 | 6 | - | Arabidopsis thaliana | Light |  |
| CaHDZ34 | GT1-motif | GGTTAAT | 1334 | 7 | + | Avena sativa | Light |  |
| CaHDZ34 | MRE | AACCTAA | 54 | 7 | - | Petroselinum crispum | MYB binding site | |
| CaHDZ34 | ARE | AAACCA | 1 | 6 | - | Zea mays | Anaerobic induction | |
| CaHDZ34 | ARE | AAACCA | 616 | 6 | - | Zea mays | Anaerobic induction | |
| CaHDZ34 | ARE | AAACCA | 640 | 6 | + | Zea mays | Anaerobic induction | |
| CaHDZ34 | ARE | AAACCA | 1178 | 6 | + | Zea mays | Anaerobic induction | |
| CaHDZ34 | LTR | CCGAAA | 552 | 6 | + | Hordeum vulgare | Low temperature | |
| CaHDZ34 | LTR | CCGAAA | 579 | 6 | + | Hordeum vulgare | Low temperature | |
| CaHDZ34 | MBS | CAACTG | 1224 | 6 | - | Arabidopsis thaliana | MYB binding site | |
| CaHDZ34 | MBS | CAACTG | 1256 | 6 | + | Arabidopsis thaliana | MYB binding site | |
| CaHDZ35 | Box 4 | ATTAAT | 622 | 6 | + | Petroselinum crispum | Light |  |
| CaHDZ35 | Box 4 | ATTAAT | 702 | 6 | + | Petroselinum crispum | Light |  |
| CaHDZ35 | Box 4 | ATTAAT | 953 | 6 | + | Petroselinum crispum | Light |  |
| CaHDZ35 | Box 4 | ATTAAT | 984 | 6 | + | Petroselinum crispum | Light |  |
| CaHDZ35 | CCAAT-box | CAACGG | 1485 | 6 | - | Hordeum vulgare | MYB binding site | |
| CaHDZ35 | TCT-motif | TCTTAC | 1639 | 6 | - | Arabidopsis thaliana | Light |  |
| CaHDZ36 | P-box | CCTTTTG | 1717 | 7 | - | Oryza sativa | Gibberellin | |
| CaHDZ36 | ACA-motif | AATCACAACCATA | 968 | 12 | + | Arabidopsis thaliana | Light |  |
| CaHDZ36 | AE-box | AGAAACTT | 522 | 8 | + | Arabidopsis thaliana | Light |  |
| CaHDZ36 | G-Box | CACGTT | 1403 | 6 | - | Pisum sativum | Light |  |
| CaHDZ36 | GT1-motif | GGTTAA | 529 | 6 | - | Arabidopsis thaliana | Light |  |
| CaHDZ36 | ARE | AAACCA | 503 | 6 | + | Zea mays | Anaerobic induction | |
| CaHDZ36 | ARE | AAACCA | 800 | 6 | + | Zea mays | Anaerobic induction | |
| CaHDZ36 | ARE | AAACCA | 1496 | 6 | - | Zea mays | Anaerobic induction | |
| CaHDZ36 | ARE | AAACCA | 1641 | 6 | - | Zea mays | Anaerobic induction | |
| CaHDZ36 | ARE | AAACCA | 1713 | 6 | + | Zea mays | Anaerobic induction | |
| CaHDZ36 | LTR | CCGAAA | 1381 | 6 | + | Hordeum vulgare | Low temperature | |
| CaHDZ36 | MBS | CAACTG | 1219 | 6 | + | Arabidopsis thaliana | MYB binding site | |
| CaHDZ36 | GC-motif | CCCCCG | 200 | 6 | - | Zea mays | Anoxic specific inducibility | |
| CaHDZ36 | TCT-motif | TCTTAC | 519 | 6 | - | Arabidopsis thaliana | Light |  |
| CaHDZ36 | ABRE | ACGTG | 1404 | 5 | + | Arabidopsis thaliana | Abscisic acid | |
| CaHDZ40 | CGTCA-motif | CGTCA | 1270 | 5 | + | Hordeum vulgare | MeJA |  |
| CaHDZ40 | MBSI | aaaAaaC(G/C)GTTA | 1018 | 10.5 | + | Petunia hybrida | MYB binding site | |
| CaHDZ40 | MBSI | aaaAaaC(G/C)GTTA | 1165 | 10.5 | + | Petunia hybrida | MYB binding site | |
| CaHDZ40 | AE-box | AGAAACAA | 1797 | 8 | - | Arabidopsis thaliana | Light |  |
| CaHDZ40 | GARE-motif | TCTGTTG | 1259 | 7 | + | Brassica oleracea | Gibberellin | |
| CaHDZ40 | Box 4 | ATTAAT | 411 | 6 | + | Petroselinum crispum | Light |  |
| CaHDZ40 | Box 4 | ATTAAT | 499 | 6 | + | Petroselinum crispum | Light |  |
| CaHDZ40 | Box 4 | ATTAAT | 763 | 6 | + | Petroselinum crispum | Light |  |
| CaHDZ40 | Box 4 | ATTAAT | 980 | 6 | + | Petroselinum crispum | Light |  |
| CaHDZ40 | Box 4 | ATTAAT | 1058 | 6 | - | Petroselinum crispum | Light |  |
| CaHDZ40 | TCCC-motif | TCTCCCT | 1762 | 7 | + | Spinacia oleracea | Light |  |
| CaHDZ40 | TGACG-motif | TGACG | 1270 | 5 | - | Hordeum vulgare | MeJA |  |
| CaHDZ40 | GTGGC-motif | CAGCGTGTGGC | 1486 | 10 | - | Hordeum vulgare | Light |  |
| CaHDZ40 | TATC-box | TATCCCA | 427 | 7 | + | Oryza sativa | Gibberellin | |
| CaHDZ40 | chs-CMA1a | TTACTTAA | 34 | 8 | + | Daucus carota | Light |  |
| CaHDZ40 | G-Box | CACGTG | 196 | 6 | + | Pisum sativum | Light |  |
| CaHDZ40 | G-box | CACGTG | 196 | 6 | + | Arabidopsis thaliana | Light |  |
| CaHDZ40 | GT1-motif | GGTTAAT | 317 | 7 | + | Avena sativa | Light |  |
| CaHDZ40 | MRE | AACCTAA | 1660 | 7 | + | Petroselinum crispum | MYB binding site | |
| CaHDZ40 | ARE | AAACCA | 73 | 6 | + | Zea mays | Anaerobic induction | |
| CaHDZ40 | MBS | CAACTG | 1466 | 6 | - | Arabidopsis thaliana | MYB binding site | |
| CaHDZ40 | TCT-motif | TCTTAC | 6 | 6 | - | Arabidopsis thaliana | Light |  |
| CaHDZ40 | TCT-motif | TCTTAC | 137 | 6 | - | Arabidopsis thaliana | Light |  |
| CaHDZ40 | ABRE | CACGTG | 196 | 6 | + | Arabidopsis thaliana | Abscisic acid | |
| CaHDZ40 | ABRE | ACGTG | 197 | 5 | + | Arabidopsis thaliana | Abscisic acid | |
