# Supplementary material for: Genome-Wide Characterization and Expression Analysis of the HD-ZIP Gene Family in Response to Salt Stress in Pepper
Source: Int J Genomics. 2021 Jan 25;2021:8105124. doi: 10.1155/2021/8105124 (PMC7869415; doi:10.1155/2021/8105124)
Supplement: Supplementary 7 — Table S7: correlation between HD-ZIP gene RNA-seq data and qRT-PCR of 9 pepper. [file 8105124.f7.docx]

**The Foldchange values of 4h and 58h under salt stress**

|  | 4h | | 58h | |
| --- | --- | --- | --- | --- |
| Gene ID | RNA-seq | qRT-PCR | RNA-seq | qRT-PCR |
| CaHDZ03 | 1.9917 | 4.01136 | 3.1227 | 4.376698 |
| CaHDZ04 | 0.66055 | 0.628919 | 0.1119 | 0.059469 |
| CaHDZ10 | -0.11924 | -0.42359 | 0.2478 | 0.157355 |
| CaHDZ21 | -0.52384 | -0.43918 | -0.31151 | -0.46262 |
| CaHDZ25 | 0.35588 | 0.405317 | 0.29975 | 0.321776 |
| CaHDZ32 | 0.1649 | 0.172554 | -0.51399 | -0.18548 |
| CaHDZ33 | -0.18689 | -0.05745 | -0.85963 | -0.40051 |
| CaHDZ35 | 0.83996 | 0.621313 | 0.17814 | 0.126667 |
| CaHDZ39 | 0.007232 | 0.423234 | 0.027255 | 0.021814 |

| 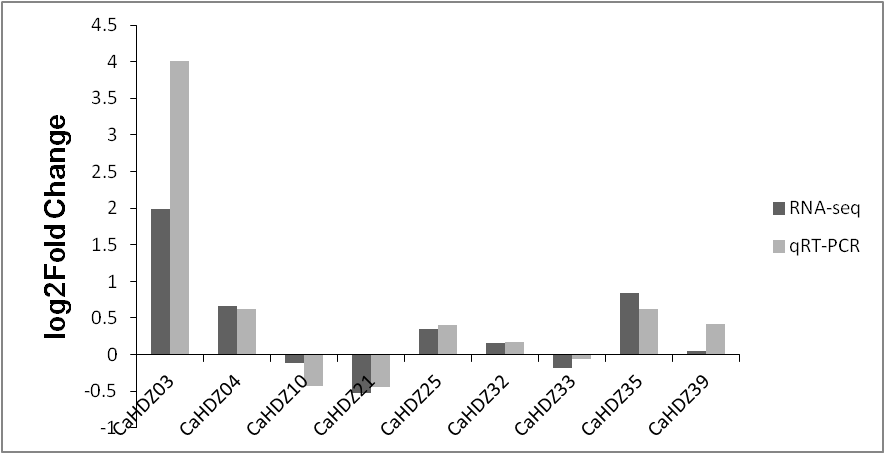 |
| --- |
| 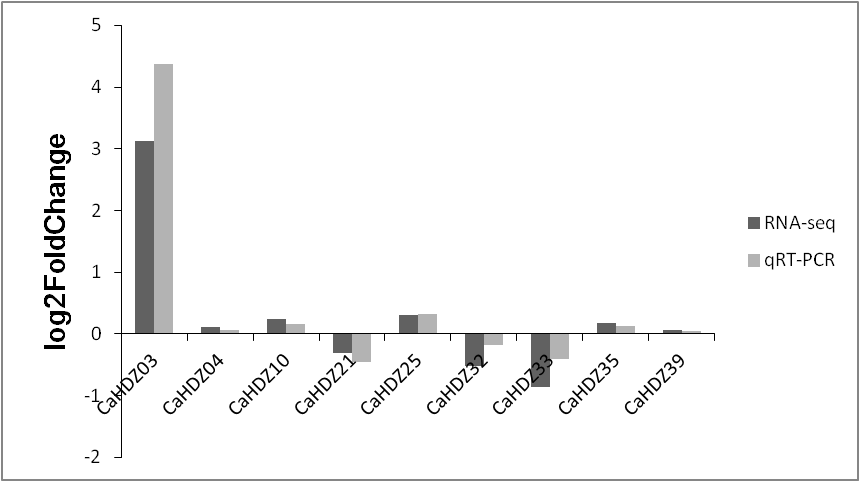 |
| 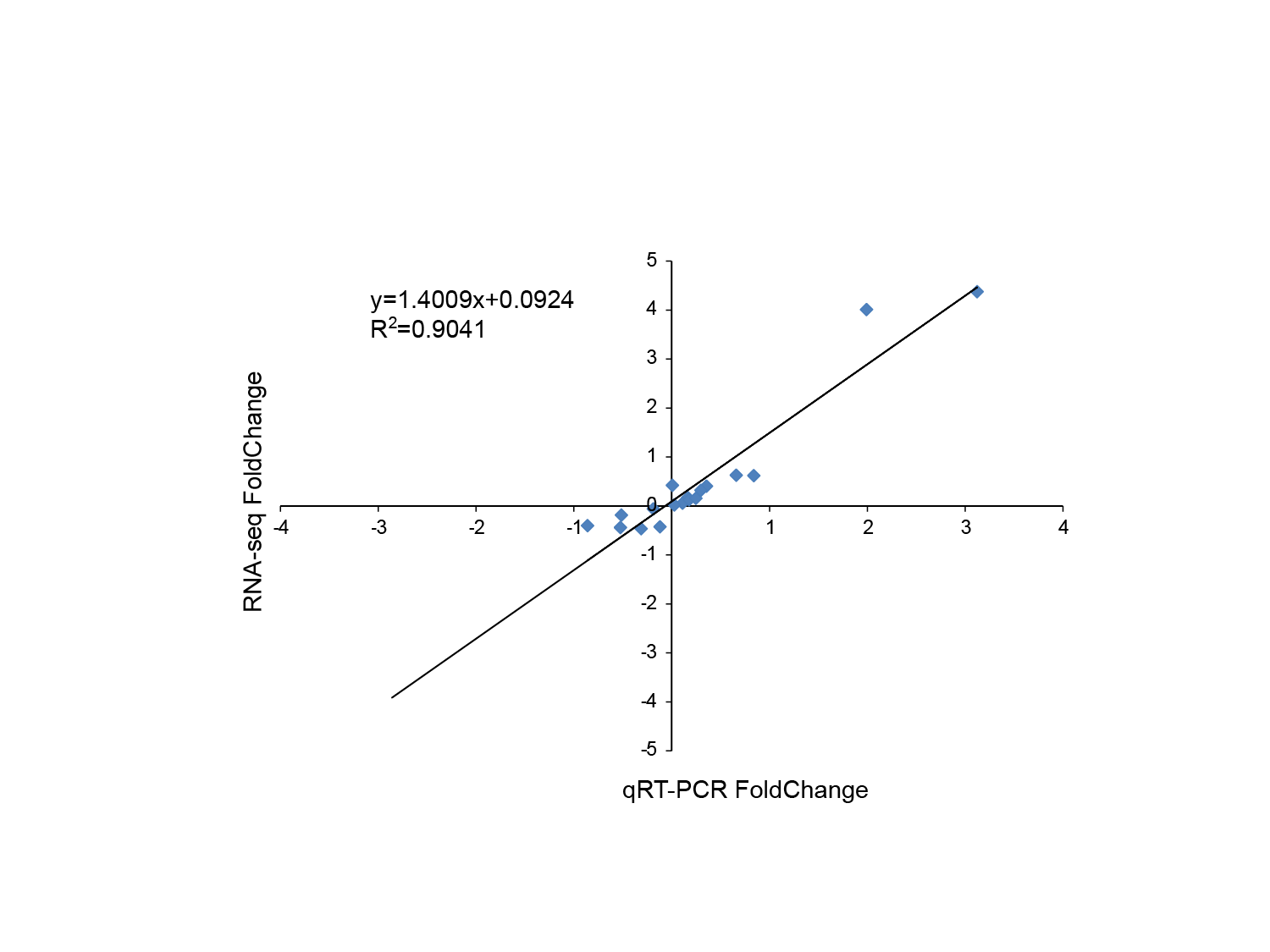 |

S7 Correlation between HD-ZIP gene RNA-seq data and qRT-PCR of 9 peppers.
